# Geminin overexpression-dependent recruitment and crosstalk with mesenchymal stem cells enhance aggressiveness in triple negative breast cancers

**DOI:** 10.18632/oncotarget.8029

**Published:** 2016-03-10

**Authors:** Suryatheja Ananthula, Abhilasha Sinha, Mohamed El Gassim, Simran Batth, Gailen D. Marshall, Lauren H. Gardner, Yoshiko Shimizu, Wael M. ElShamy

**Affiliations:** ^1^ Cancer Institute, University of Mississippi Medical Center, Jackson, MS, USA; ^2^ Department of Medicine, University of Mississippi Medical Center, Jackson, MS, USA; ^3^ Institute for Biogenesis Research, Cancer Center, University of Hawaii, Honolulu, HI, USA; ^4^ Clinical and Translational Research Program, Cancer Center, University of Hawaii, Honolulu, HI, USA

**Keywords:** breast cancer, triple negative, geminin, c-Abl, HMGB1

## Abstract

Resident mesenchymal stem cells (MSCs) promote cancer progression. However, pathways and mechanisms involved in recruiting MSCs into breast tumors remain largely undefined. Here we show that geminin-dependent acetylation releases HMGB1 from the chromatin to the cytoplasm and extracellular space. Extracellular acetylated HMGB1 (Ac-HMGB1) promotes geminin overexpressing (GemOE) cells survival by binding to RAGE and activating NF-κB signaling. Extracellular Ac-HMGB1 also triggers expression and activation of RAGE in the non-expressing MSCs. RAGE activation induces expression of CXCR4 in MSCs and directional migration towards SDF1 (*aka* CXCL12)-expressing GemOE cells *in vitro* and *in vivo*. These effects augmented by the necrotic and hypoxic environment in GemOE tumors, especially within their cores. Reciprocal interactions between newly recruited MSCs and GemOE tumor cells elevate tumor-initiating (TIC), basal and epithelial-to-mesenchymal transition (EMT) traits and enhance aggressiveness *in vitro* and *in vivo* in GemOE tumor cells. Indeed, faster, larger and more aggressive tumors develop when GemOE cells are co-injected with MSCs in orthotopic breast tumor model. Concurrently, inhibiting c-Abl (and thus geminin function), RAGE or CXCR4 prevented MSCs recruitment to GemOE cells *in vitro* and *in vivo*, and decreased the TIC, basal and EMT phenotypes in these tumor cells. Accordingly, we propose that GemOE tumor cells present within tumor cores represent metastatic precursors, and suppressing the GemOE→HMGB1/RAGE→SDF1/CXCR4 signaling circuit could be a valid target for therapies to inhibit GemOE tumors and their metastases.

## INTRODUCTION

Mesenchymal stem cells (MSCs) are heterogeneous type of cells dispatched from bone marrow to different organs to maintain tissue homeostasis [1]. MSCs can be actively recruited into tumors in a migration mode driven by a specific repertoire of soluble factors [2, 3]. Prominent among these factors is the stromal differentiation factor (SDF1, *aka* CXCL12) [4, 5]. Once inside the tumor, MSCs through bi-directional interactions enhance tumor cells invasion and metastatic capabilities [6].

In highly proliferative solid tumors, due to increased proximity to vessels and neo-angiogenesis, e.g., in tumor cores, hypoxia ensues [7]. Hypoxia promotes both resistance to conventional cancer therapies and tumor progression by creating microenvironment enriched in poorly differentiated tumor cells and undifferentiated stromal cells, including MSCs [7], in part, through stabilization of the transcription activator, “hypoxia-inducible factor-1 alpha (HIF-1α)” in tumor and stromal cells [8].

Within tumor cores many cells also die by necrosis and passively release intracellular alarmins (*aka*, damage-associated molecular patterns or DAMPs). Prominent among these DAMPs is the high mobility group B1 “HMGB1”. HMGB1 is a nuclear protein with high affinity to DNA but no sequence preference involved in replication, transcription and recombination among other functions [9]. HMGB1 functions also extend beyond the nucleus. Released from certain cells, including cancer cells, HMGB1 plays important roles in inflammation and tumor metastasis [10, 11]. Post-translational modifications determine HMGB1 functions and release mechanisms [12]. Indeed, mono-methylated on lysine 43 (K43) HMGB1 is released from neutrophils. Cysteine redox isoforms of HMGB1 act as both a chemotactic and a cytokine-inducing mediator [12]. Acetylation seems to be the major modification affecting HMGB1 localization. Hyper-acetylation within the nuclear localization sequence 2 (NLS2) by CBP promotes HMGB1 dissociation from chromatin, decreases the level within the nucleus and increases secretion [13–15]. Conversely, de-acetylation by SIRT1 at K55, K88, K90, and K177 within the pro-inflammatory and NLS2 domains prevents HMGB1 cytoplasmic localization [15, 16]. Interestingly, genetic ablation or pharmacological inhibition of SIRT1 in endothelial cells reduced HMGB1 nuclear localization, enhanced cytoplasmic translocation and promoted secretion [16]. Conversely, resveratrol an activator of SIRT1 decreased HMGB1 acetylation thereby increasing nuclear retention [13]. Moreover, inflammation, a known suppressor of SIRT1 *in vivo*, activates HMGB1 acetylation, cytoplasmic translocation, and systemic release thereby maintains inflammation [15]. Extracellular HMGB1 signals through binding to receptor for advanced glycation end products (RAGE) or toll-like receptors (TLR) expressed on the surface of many cells including monocytes/macrophages, T-lymphocytes, neurons, endothelial cells, osteoclasts/osteoblasts, mesenchymal stem cells and a variety of tumor cells [17–19]. Activated RAGE signals by activating NF-κB-mediating inflammatory gene expression [20–21], adding support to the connection between chronic inflammation and cancer progression [22]. Indeed, pharmacological inactivation of RAGE shows great clinical efficacy in pre-clinical tumor mouse models [23].

Geminin overexpressing breast cancer cells overexpress a nuclear only form of c-Abl [24], a phenotype we now refer to as “GemOE”. c-Abl phosphorylation of geminin tyrosine (Y) 150 stabilizes the protein [24] and activates geminin oncogenic function, *in vitro* and *in vivo* [24-26]. Inhibiting Y150 phosphorylation destabilizes geminin protein leading to death of GemOE cells specifically, with no effect on low geminin and cytoplasmic c-Abl-expressing normal human mammary epithelial (HME) cells [25]. *In vivo*, GemOE tumors are extremely sensitive to c-Abl inhibitors; e.g., imatinib and nilotinib [24]. Moreover, because GemOE is detected in more than half of triple negative breast cancers (TNBCs), we recently proposed the use of imatinib or nilotinib to treated TNBC patients re-stratified according to the GemOE criterion [24].

In the current study, we show geminin-dependent acetylation and extracellular secretion of chromatin-bound HMGB1 from necrotic/hypoxic GemOE in tumor cores. Secreted Ac-HMGB1 in autocrine/paracrine fashion promotes survival of GemOE/TNBC tumor cells. Extracellular Ac-HMGB1 also stimulates expression and activation of RAGE on the surface of the non-expressing MSCs. In MSCs, activated RAGE triggers CXCR4 expression leading to directed migration of MSCs towards SDF1-secreting GemOE/TNBC cells in tumor cores. In tumor cores, reciprocal interactions between MSCs and GemOE/TNBC cells stimulate aggressiveness in GemOE/TNBC tumor cells. Accordingly, inhibiting geminin Y150 phosphorylation (i.e. using imatinib), HMGB1 secretion or binding to RAGE or CXCR4 activity inhibited survival of GemOE tumor cells, recruitment of MSCs *in vitro* and *in vivo* into GemOE/TNBC tumors’ core and significantly reduced the aggressive traits of GemOE/TNBC cells.

## RESULTS

### Geminin, HMGB1 complex formation

**Figure 1:**
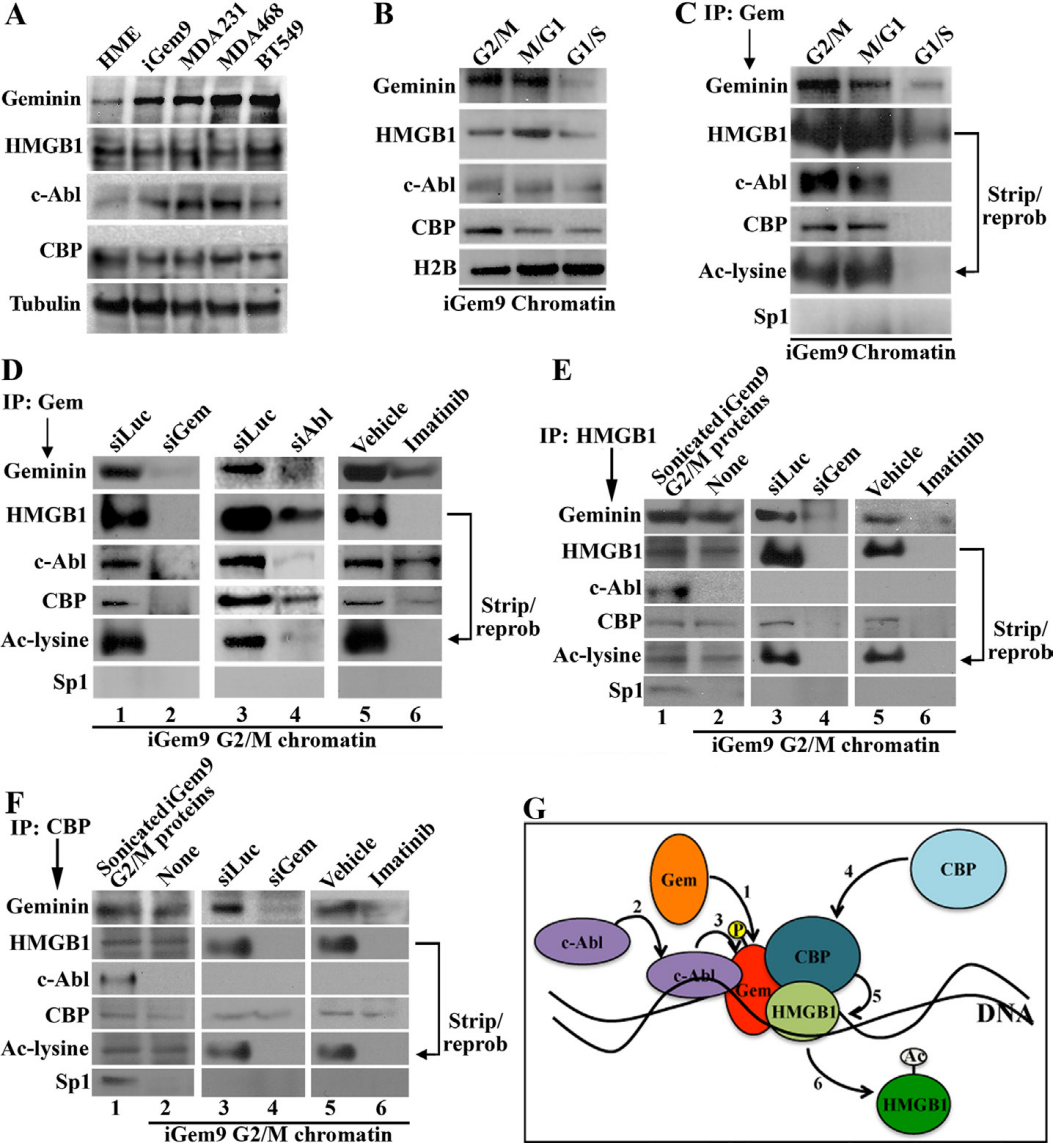
Geminin promotes acetylation of chromatin-bound HMGB1 (**A**) The levels of indicated proteins in naïve HME, iGem9 cells (defined as Dox-induced for 72 h) and the TNBC cell lines MDA-MB-231, MDA-MB-468 and BT549 sonicated extracts. (**B**) The levels of indicated proteins on the chromatin of iGem9 cells synchronized in G_2_/M, M/G_1_ and G_1_/S phases. (**C**) IP experiments using geminin specific antibody on chromatin isolated from G_2_/M, M/G_1_ and G_1_/S phase iGem9 cells and blotted for the indicated proteins. (**D**) IP experiments using geminin specific antibody on G_2_/M phase iGem9 chromatin extracts of cells transfected with siLuc, siGem or siAbl for 72 h or treated with vehicle or 10 μM imatinib for 24 h. IP experiments using HMGB1 specific antibody (**E**) or CBP specific antibody (**F**) on iGem9 cells synchronized in G_2_/M-phase sonicated extracts (first lanes), or chromatin extracts following 72 h of transfection with siLuc or siGem or 24 h treatment with vehicle or 10 μM imatinib. (**G**) Schematic representation of the data presented through out this figure. In all parts of the figure experiments were done between 2–3 separate times.

In a yeast 2-hybrid screen with full-length geminin as bait, we recently identified HMGB1 as a binding partner. Total proteins from naïve mammary epithelial (HME) cells, inducible Gem9 (iGem9, a HME cell line expressing a doxycycline [Dox]-inducible geminin allele) for at least 72 h and three TNBC cell lines, MDA-MB-231, MDA-MB-468 and BT549 (endogenously overexpressing geminin), were isolated by sonication. Geminin level is low in naïve HME but high in iGem9 cells to a level that resembles that of the TNBC cell lines (Figure 1A). In contrast, HMGB1 level was similar in all cell lines, including naïve HME cells (Figure 1A). Quantitatively, compared to naïve HME cells, iGem9 and TNBC cell lines express 5–6 fold higher geminin but similar levels of HMGB1 (Supplementary Figure 1A). In accordance, total cell extract c-Abl level is higher in iGem9 and TNBC cell lines compared to naïve HME cells (Figure 1A and Supplementary Figure 1A), while CBP is expressed at similar level (Figure 1A and Supplementary Table 1A).

Geminin resides in different nuclear compartments in cell cycle-dependent manner. In late G_1_ and S phases, geminin is a nuclear soluble protein, whereas in G_2_/M/early G_1_ phases it becomes chromatin bound protein [27]. To determine the level on the chromatin in different phases of the cell cycle, G_2_/M, M/G_1_ or G_1_/S phase chromatin was isolated from iGem9 cells. Geminin, HMGB1 and c-Abl levels were highest on G_2_/M and M/G_1_ phase chromatin, and significantly dropped in G_1_/S cells chromatin (Figure 1B and Supplementary Table 1B). CBP level was highest in G_2_/M-phase cells chromatin, dropped slightly in M/G_1_-phase cells chromatin and dropped further in G_1_/S-phase cells chromatin (Figure 1B, Supplementary Figure 1B). Together suggests that the 4 proteins are present on the chromatin during G_2_/M and M/G_1_ phase cells, but not on G_1_/S phase cells' chromatin.

To confirm the putative interaction identified in the 2-hybrid screen, G_2_/M-, M/G_1_- and G_1_/S-phases iGem9 cells chromatin extracts were immunoprecipitated (IPd) using a monoclonal anti-geminin antibody. Western blot analysis of these IPs showed that geminin antibody pulled-down c-Abl, CBP and HMGB1, which was acetylated as detected by stripping and re-probing using anti-Ac-lysine antibody from G_2_/M (1st lane Figure 1C) and M/G_1_ (2nd lane Figure 1C) cells chromatin. In contrast, in G_1_/S phase low-level HMGB1 only was pulled-down with geminin antibody that was not acetylated (3rd lane Figure 1C). Together suggests that a complex between geminin and HMGB1 together with c-Abl and CBP presences on the chromatin of G_2_/M and M/G_1_ GemOE cells is perhaps involved in acetylation of HMGB1.

### Phosphorylation of HMGB1-bound geminin on the chromatin in G_2_ phase

Previous studies clearly showed that cell-cycle dependent modifications play important roles in the localization and function of HMGB1 [13–16] and geminin [24–27]. Since the data presented above suggests a complex formation predominantly in G_2_/M-phase, iGem9 cells were first transfected with luciferase siRNA (siLuc, negative control), sigeminin (siGem) or siAbl for 72 h. Another set of cells was instead treated with vehicle or 10 μM imatinib for 24 h. Chromatin extracts isolated from all these cells synchronized in G_2_/M phase were IPd using anti-geminin, -HMGB1 or -CBP antibodies. First, in control treated cells, anti-geminin IPd c-Abl, CBP and HMGB1 that was acetylated (1st, 3rd and 5th lanes in Figure 1D). In support of our previous results [24, 25], geminin silencing (2nd lane in Figure 1D), c-Abl silencing (4th lane in Figure 1D) or inactivation (6th lane in Figure 1D) significantly reduced geminin on the chromatin (2nd, 4th and 6th lanes in Figure 1D), which according to the above data led to significant decrease in the level of HMGB1 (and Ac-HMGB1) and CBP co-IPd (2nd, 4th and 6th lanes in Figure 1D). Furthermore, although all proteins were present in G_2_/M phase cells chromatin (1st lanes in Figure 1E and 1F), HMGB1 or CBP antibodies co-IPd all components of the complex except c-Abl (2nd, 3rd and 5th lanes in Figure 1E and 1F). Geminin silencing or c-Abl inactivation (hence geminin depletion) disassembled the complex as detected by lack of pull-down of all proteins in the anti-HMGB1 IPs (4th and 6th lanes in Figure 1E) or anti-CBP IPs (4th and 6th lanes in Figure 1F). Taken together suggests that geminin translocates to the chromatin (see also [27]) perhaps at HMGB1 sites in late S/early G_2_ phase (step 1 in Figure 1G). Since yeast cells do not modify proteins the same way human cells do, our yeast 2-hybrid screen data suggests that this interaction perhaps initially occurs between HMGB1 and a non-phosphorylated geminin. c-Abl is then recruited to this complex to phosphorylate geminin (step 2 and 3 in Figure 1G), which perhaps leads to recruitment of CBP (step 4 in Figure 1G) to the complex to acetylate HMGB1 (step 5 in Figure 1G). CBP binding seems transient, leaving after HMGB1 acetylation, perhaps due to change in geminin phosphorylation status from Y-phosphorylated to serine/threonine-phosphorylated occurring in late M/early G_1_−phase and/or geminin release from chromatin also during this phase [27]. Acetylated HMGB1 (Ac-HMGB1) is most likely released from the chromatin (step 6 in Figure 1G) [13, 14]. Lack of geminin seems to prevent complex formation, HMGB1 acetylation and release.

### Rapid secretion of cytoplasmic Ac-HMGB1 in GemOE cells

**Figure 2:**
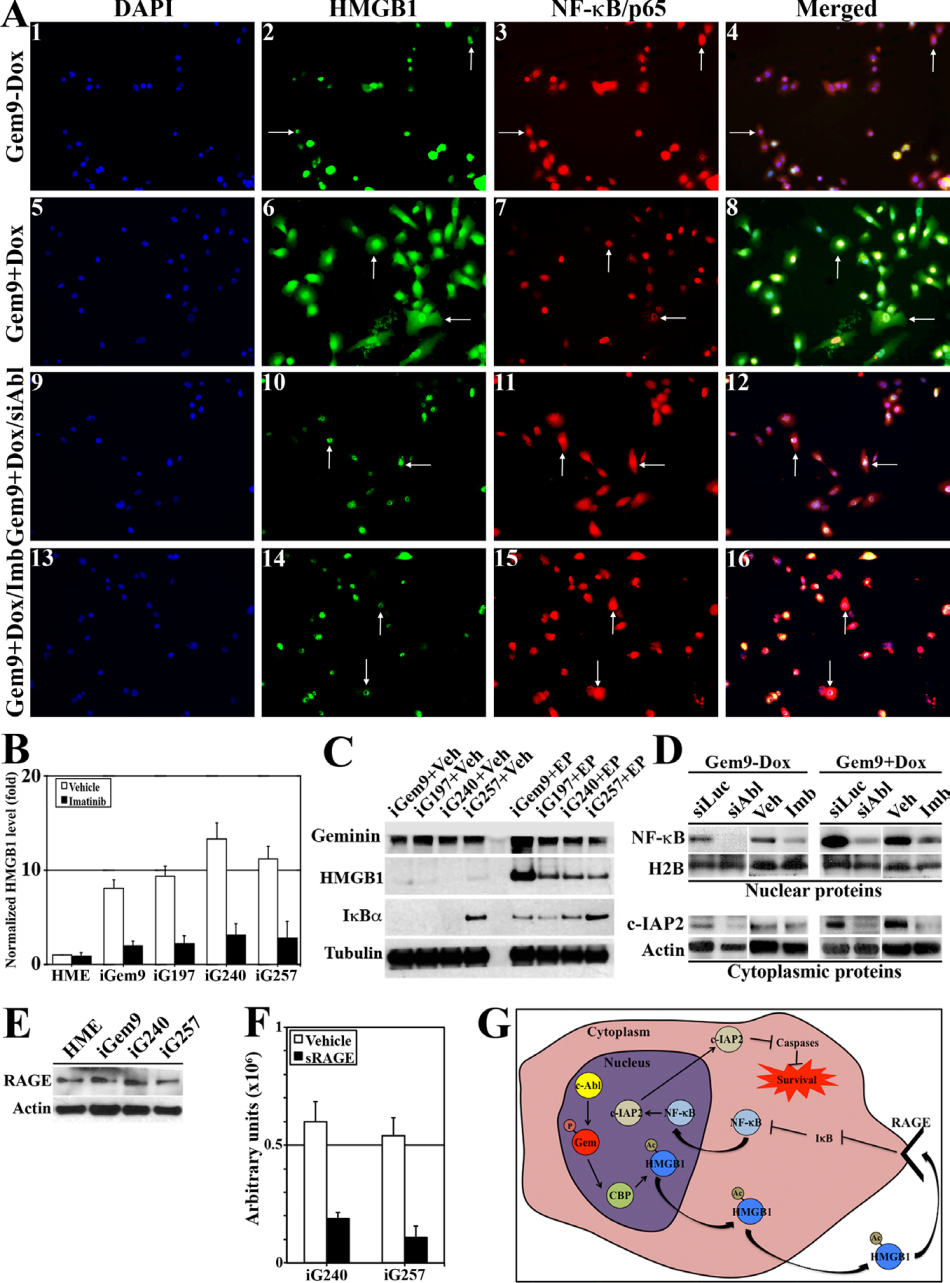
Geminin overexpression enhances HMGB1 acetylation, release from chromatin, cytoplasmic translocation and secretion (**A**) Immunofluorescence analysis of HMGB1 and NF-κB/p65 in uninduced Gem9 (1–4) or iGem9 cells before (5–8) or after c-Abl silencing for 72 h (9–12) or inactivation using 10 μM imatinib for 24 h (13–16). (**B**) The levels of HMGB1 in CM of naïve HME, iGem9, iG197, iG240 and iG257 cells in the presence of vehicle (white bars) or 10 μM imatinib (black bars) for 24 h detected using specific HMGB1 ELISA assay. Experiments were done in triplicates 3 different times, **represents *p < 0.001*. (**C**) The levels of HMGB1 and the endogenous inhibitor of NF-κB; IκBα in the cytoplasm of iGem9, iG197, iG240 and iG257 cells in the absence (Veh, left) or the presence (200 μM EP, right) of the inhibitor of HMGB1 release; ethyl pyruvate. (**D**) The levels of active NF-κB/p65 and the survival factor c-IAP2 in the cytoplasmic or chromatin fraction of uninduced Gem9 cells or iGem9 cells transfected with siLuc or siAbl for 72 h or treated with vehicle or 10 μM imatinib for 24 h. (**E**) RAGE levels in naïve HME, iGem9, iG240 or iG257 cells. (**F**) Activation of NF-κB response element in iG240 or iG257 cells following vehicle or 10 μg/ml sRAGE for 24 h. Experiments were done in triplicates 3 different times, **represents *p < 0.001.* (**G**) Schematic representation of the data presented throughout this figure. In all parts of the figure experiments were done between 2–3 separate times.

To determine the localization of Ac-HMGB1 in GemOE cells, uninduced-Gem9 or iGem9 cells were stained with anti-HMGB1 and -NF-κB/p65 (a target of cytoplasmic HMGB1) [28, 29] antibodies. In uninduced Gem9 cells (Figure 2A_1–4_), HMGB1 was predominantly nuclear (arrows in Figure 2A_2_) and NF-κB/p65 was cytoplasmic (i.e. inactive, arrows in Figure 2A_3_). In contrast, in iGem9 (arrows in Figure 2A_5–8_) increased cytoplasmic HMGB1 (arrows in Figure 2A_6_) and nuclear/active NF-κB/p65 (arrows in Figure 2A_7_) was detected. c-Abl silencing (arrows in Figure 2A_9–12_) or inactivation (arrows in Figure 2A_13–16_) in iGem9 cells led to HMGB1 re-localization to the nucleus (arrows in Figure 2A_10_ and 2A_14_) and NF-κB/p65 to the cytoplasm (arrows in Figure 2A_11_ and 2A_15_).

In addition to Gem9, we previously generated several inducible geminin expressing cell lines. Five of these cell lines (including Gem9) were injected (5 × 10^6^ cells/mouse) orthotopically in SCID mice mammary fat pads (*n* = 5/cell line) and mice were maintained on Dox-water. Tumors (i.e. GemOE-driven mammary tumors) developed were isolated and used to generate cell lines. In the following experiments, three cell lines now named: G197, G240 and G257 that were generated by a different parental cell line and still maintained on Dox *in vitro* and *in vivo* will be used in addition to the parental Gem9 to perform the following analysis.

Naïve HME, Gem9, G197, G240 and G257 cells induced for 72 h were grown in the presence of vehicle or 10 μM imatinib for an additional 24 h. Using a specific HMGB1 ELISA, we compared the level of HMGB1 secreted from these cells. Compared to naïve HME cells conditioned medium (CM), iGem9, iG197, iG240 and iG257 cells CM contained 8_–_14 fold higher HMGB1 (white bars in Figure 2B), reinforcing the notion that geminin overexpression triggers HMGB1 acetylation and secretion. Imatinib treatment had insignificant effect on the amount of HMGB1 secreted from naïve HME cells, but significantly reduced the amount of HMGB1 secreted from iGem9, iG197, iG240, iG257 cell lines (black bars in Figure 2B). To confirm that further, iGem9, iGem197, iG240 and iG257 cells were treated with ethyl pyruvate (EP), an aliphatic ester derived from pyruvic acid, that inhibits HMGB1 secretion [30]. While in vehicle (Veh) treated iGem9, iG197, iG240, iG257 cells, there was virtually no cytoplasmic HMGB1 (2nd panel in Figure 2C, left), treatment with 200 μM EP for 24 h led to significant accumulation of HMGB1 in the cytoplasm of all cell lines (2nd panel in Figure 2C, right). Taken together confirms that in GemOE cells, HMGB1 is predominantly acetylated and translocated to the cytoplasm in a transient manner before it is secreted.

### HMGB1 secreted from GemOE cells activates NF-κB signaling in GemOE cells

It is well known that HMGB1 signal through NF- κB [22]. IκBα binds and sequesters NF-κB in an inactive cytoplasmic form in many cell types [29]. Cell activation leads to IκBα phosphorylation and degradation leading to release of NF-κB and translocation into the nucleus to activate expression of several survival genes [28, 29]. Compared to Veh-treated cells that showed almost complete absence of cytoplasmic IκBα (3rd panel in Figure 2C, left), EP-treated cells retained high levels of cytoplasmic IκBα (3rd panel in Figure 2C, right). This suggests that in auto/paracrine fashion secreted HMGB1 activates NF-κB in GemOE cells.

To confirm that nuclear and cytoplasmic proteins from uninduced-Gem9 or iGem9 cells (for 72 h) in which c-Abl was either silenced (for 72 h) or inactivated (using 10 μM imatinib, for 24 h) were isolated. Compared to uninduced-Gem9 cells that showed low level of nuclear NF-κB/p65 and low level of cytoplasmic c-IAP2 (a transcription target of activated NF-κB), iGem9 cells showed high levels of nuclear NF-κB/p65 and cytoplasmic c-IAP2 (compare 5th and 7th to 1st and 3rd lanes in Figure 2D). c-Abl silencing or inactivation significantly reduced the levels of nuclear NF-κB/p65 and cytoplasmic c-IAP2 in uninduced-Gem9 (compare 2nd to 1st lane and 4th to 3rd lane, respectively in Figure 2D) as well as iGem9 cells (compare 6th to 5th lane and 8th to 7th lane, respectively in Figure 2D).

Secreted Ac-HMGB1 can signal through binding to RAGE or TLR to activate NF-κB signaling in many cell types [21, 31]. To study which of these receptors is involved in enhancement of GemOE cell survival induced by secreted Ac-HMGB1, membrane proteins from naïve HME, iGem9, iG240 and iG257 were isolated. All cell lines showed almost equal levels of RAGE (Figure 2E), but complete absence of TLR4 expression on their surface (data not shown), suggesting that increase NF-κB/p65 activity following Ac-HMGB1 secretion is RAGE- and not TLR4-dependent (although other TLR could be involved). Additionally the data support the view that NF-κB activation in GemOE compared to naïve HME cells is driven by increase Ac-HMGB1 secretion and not increase RAGE expression on the surface of these cells.

To confirm this further, the highest Ac-HMGB1-secreting cell lines, iG240 and iG257 (Figure 2B) were transfected with a NF-κB-Luc reporter plasmid for 48 h. Cell were then grown in the presence or absence of 10 μg/ml of the HMGB1-RAGE uncoupling protein; soluble RAGE (sRAGE) for an additional 24 h. Compared to vehicle-treated cells, sRAGE-treated cells showed 4–5fold reduction in NF-κB reporter activity (compare black to white bars in Figure 2F). Taken together, the data clearly show that Ac-HMGB1 released from the chromatin that accumulates in the cytoplasm of GemOE cells (Figure 2A) is rapidly released (Figure 2B and 2C) to promote survival of GemOE tumor cells by activating NF-κB (Figure 2D) in an autocrine/paracrine fashion through binding to RAGE expressed on the surface of these cells (The kinetics of this is shown in Figure 2G).

### Cytoplasmic HMGB1 localization in GemOE tumor cells *in vivo*

**Figure 3:**
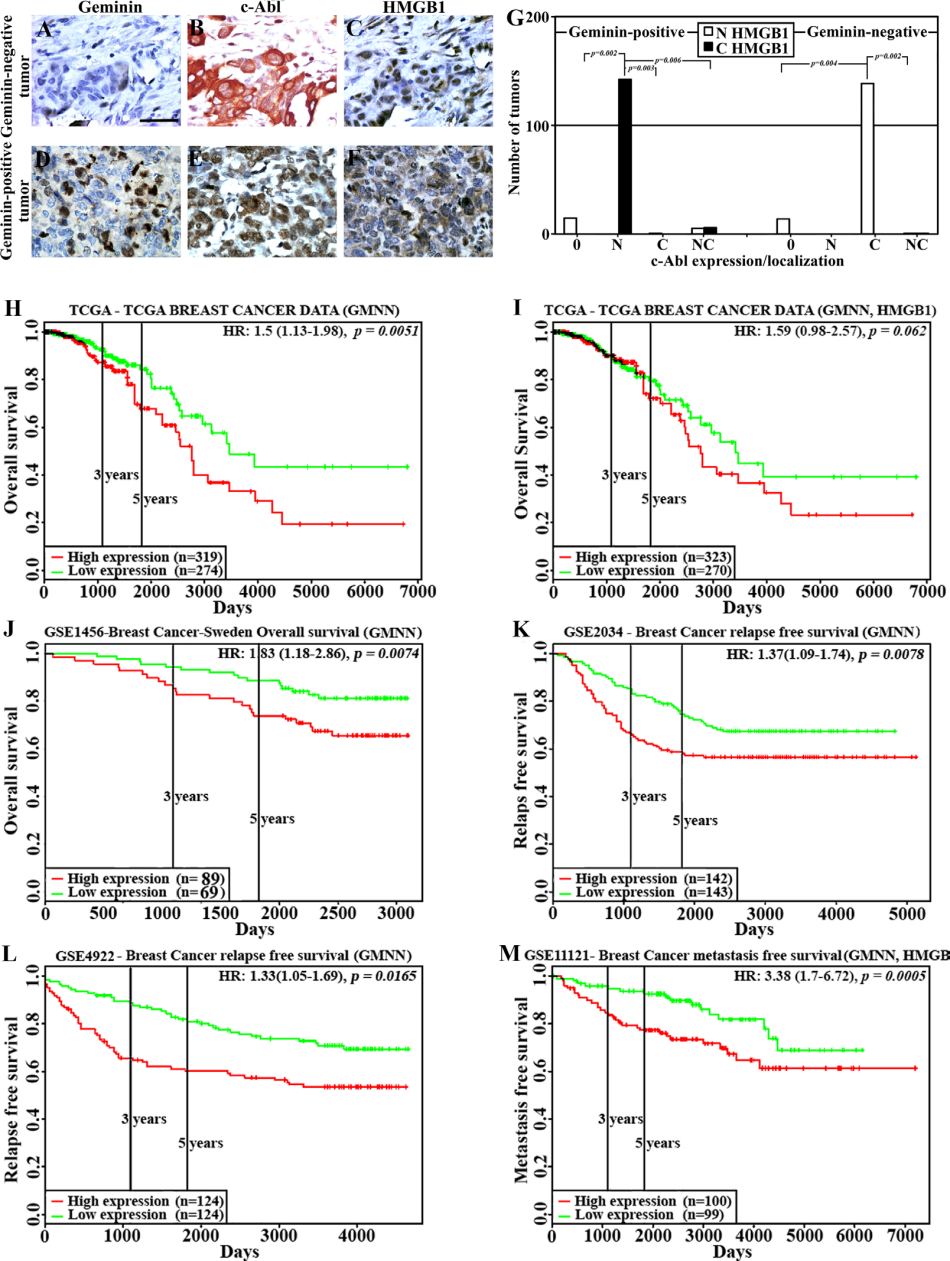
HMGB1 localization and effect on clinical outcome in GemOE tumors The expression of geminin (**A** and **D**), c-Abl (**B** and **E**) and HMGB1 (**C** and **F**) in geminin-negative (A–C) or geminin-positive (D–F) breast cancer TMAs. (**G**) Number of tumors with nuclear (white bars) or cytoplasmic (black bars) HMGB1 expression in geminin-positive (left) or geminin-negative (right) tumors subdivided according to c-Abl expression as un (0)-, nuclear (N)-, cytoplasmic (C)- or nucleo-cytoplasmic (NC)- expressing tumors. (**H**) The rate of overall survival in 593 patients from the TCGA dataset with breast tumors expressing high (red line, *n* = 319) vs. low (green line, *n* = 274) geminin with hazard ratio of 1.5 (1.13–1.98) and *p = 0.0051*. (**I**) The rate of overall survival in 593 patients from the TCGA dataset with breast tumors expressing high (red line, *n* = 323) vs. low (green line, *n* = 270) geminin + HMGB1 with hazard ratio of 1.59 (0.98–2.57) and *p = 0.062*. (**J**) The rate of overall survival in 158 patients from the GSE1456 dataset with breast tumors expressing high (red line, *n* = 89) vs. low (green line, *n* = 69) geminin with hazard ratio of 1.83 (1.18–2.86) and *p = 0.0074*. (**K**) The rate of relapse-free survival in 285 patients from the GSE2034 dataset with breast tumors expressing high (red line, *n* = 142) vs. low (green line, *n* = 143) geminin with hazard ratio of 1.37 (1.09–1.74) and *p = 0.0078*. (**L**) The rate of relapse-free survival in 248 patients from the GSE4922 dataset with breast tumors expressing high (red line, *n* = 124) vs. low (green line, *n* = 124) geminin with hazard ratio of 1.33 (1.05–1.69) and *p = 0.0165*. (**M**) The rate of metastatic-free survival in 199 patients from the GSE11121 dataset with breast tumors expressing high (red line, *n* = 100) vs. low (green line, *n* = 99) geminin with hazard ratio of 3.38 (1.7–6.72) and *p = 0.0005*.

To obtain *in vivo* confirmation of this hypothesis, a cohort of human samples consists of 66 normal/near cancer tissues and 326 breast tumors of different subtypes were analyzed using immunohistochemistry (IHC) for geminin, c-Abl and HMGB1 expression. As shown earlier [24] compared to normal/near cancer tissues, > 50% of breast tumors overexpressed geminin (geminin-positive *n* = 170 vs. geminin-negative *n* = 156). Also in accordance with our previous data [24], in geminin-negative tumors (Figure 3A), c-Abl was overexpressed as an exclusively cytoplasmic protein (Figure 3B), whereas in geminin-positive tumors (Figure 3D), c-Abl was exclusively nuclear (Figure 3E). Furthermore, although we could not detect a significant difference in the level of HMGB1 between normal and tumor tissues, it was absolutely clear that geminin-negative/cytoplasmic c-Abl-overexpressing tumors express nuclear HMGB1 (Figure 3C), whereas in total agreement with the above data, almost all geminin-positive/nuclear c-Abl-overexpressing tumors express cytoplasmic HMGB1 (Figure 3F).

To quantitate these data, geminin-positive and -negative tumors were first divided into not (0), nuclear (N), cytoplasmic (C) or nucleo-cytoplasmic (NC) c-Abl-expressing tumors, and HMGB1 expression as nuclear or cytoplasmic was analyzed in these different sub-populations. Within the geminin-positive tumors (*n* = 170), all c-Abl-negative tumors (*n* = 15) showed nuclear HMGB1 expression, all nuclear c-Abl-overexpressing tumors (*n* = 142, *p = 0.002* vs. 0, *p = 0.003* vs. C and *p = 0.006* vs. NC) showed cytoplasmic HMGB1 expression, all cytoplasmic c-Abl-overexpressing tumors (*n* = 1) showed nuclear HMGB1 expression, and from the nucleo-cytoplasmic c-Abl-overexpressing tumors (*n* = 11), 5 showed nuclear HMGB1 expression and 6 showed cytoplasmic HMGB1 expression (Figure 3G, left). In contrast, within the geminin-negative tumors (*n* = 156), all c-Abl-negative tumors (*n* = 14) showed nuclear HMGB1 expression (Figure 3G, right), there was no geminin-negative tumors overexpressing nuclear c-Abl, all cytoplasmic c-Abl-overexpressing tumors (*n* = 140, *p = 0.004* vs. 0 and *p = 0.002* vs. NC) showed nuclear HMGB1 expression (Figure 3G, right) and from the nucleo-cytoplasmic c-Abl-overexpressing tumors (*n* = 2), 1 showed nuclear HMGB1 expression and 1 showed cytoplasmic HMGB1 expression (Figure 3G, right).

### Adverse clinical outcomes in breast cancer patients overexpressing geminin

To investigate the association between geminin alone or with HMGB1 expression and clinical outcomes, “PROGgeneV2” [32, 33] resource was used to analyze publicly available datasets for the prognostic significance of increased geminin expression with or without the increase in HMGB1 expression using Kaplan-Meier method.

First, analysis of The Cancer Genome Atlas (TCGA) patients’ dataset (*n* = 593) revealed that patients with high geminin expression (*n* = 319) had significantly shorter overall survival (OS) compared to patients with low expression (*n* = 274, HR = 1.5, 95% CI = 1.13–1.98, *p* = 0.0051, Figure 3H). Moreover, in accordance with the above data showing increased geminin and not HMGB1 expression in tumor cell lines compared to naïve HME cells (Figure 1A), when the same TCGA dataset was reanalyzed instead for geminin + HMGB1 the same trend remained (i.e. geminin + HMGB1 overexpressing patients showed lower OS than low expressing HR = 1.59, 95% CI = 0.98_–_2.57) although the statistical significance somewhat decreased (*p* = 0.062, Figure 3I).

Analysis of the “GSE3494” dataset (*n* = 158 lymph-node negative (LNN) cancers resected in Uppsala, Sweden between January 1987 and December 1989) [34] for OS showed high geminin expressers (*n* = 89) had significantly shorter OS than low expressers (*n* = 69, HR = 1.83, 95% CI = 1.18_–_2.86, *p* = 0.0074, Figure 3J). Analysis of the “GSE2034” dataset (*n* = 285 LNN breast cancer patients) [35] for relapse free survival (RFS) showed that patients with high geminin expression (*n* = 142) had significantly shorter RFS than low expressing patients (*n* = 143, HR = 1.37, 95% CI = 1.09_–_1.74, *p* = 0.0078, Figure 3K). Analysis of the “GSE4922” dataset (*n* = 248 breast cancer patients) [36] for RFS showed that high geminin expressers (*n* = 124) had significantly shorter RFS than low expressers (*n* = 124, HR = 1.33, 95% CI = 1.05_–_1.69, *p* = 0.0165, Figure 3L). Finally, analysis of the “GSE11121” dataset (*n* = 199 untreated after surgery of LNN breast cancer patients [37] showed that high geminin + HMGB1 expressing patients (*n* = 100) had significantly shorter metastatic-free survival (MFS) than low expressing patients (*n* = 99, HR = 3.38, 95% CI = 1.7_–_6.72, *p* = 0.0005, Figure 3M). These data clearly show that GemOE is correlated with adverse outcomes, such as low OS and RFS in breast cancers, whereas HMGB1 seems to be involved only in increase GemOE-breast cancer metastasis.

### Hypoxia/necrosis exacerbates Ac-HMGB1 secretion from GemOE tumors *in vitro* and *in vivo*

**Figure 4:**
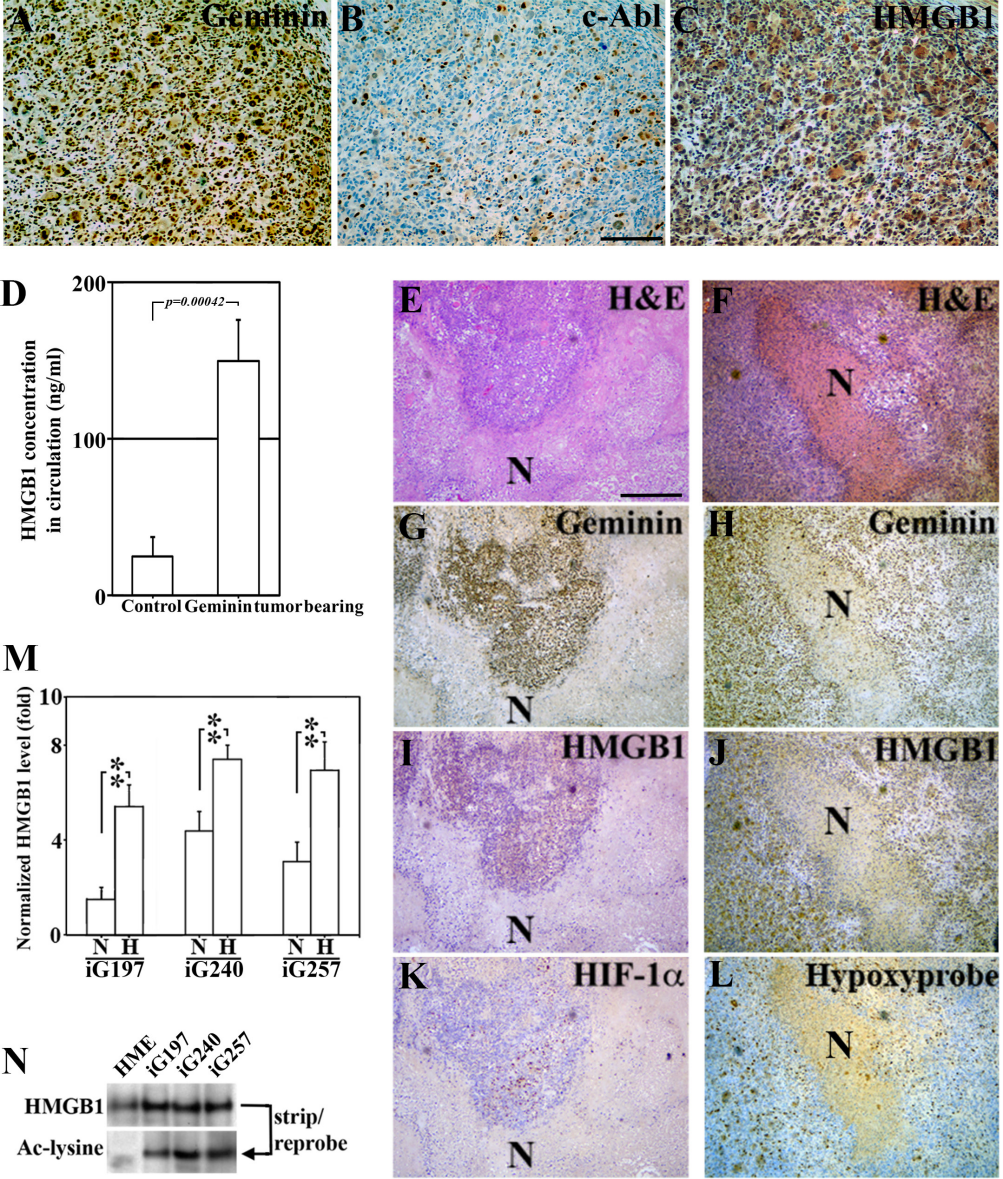
Hypoxic/necrotic core in GemOE tumors enhances Ac-HMGB1 secretion (**A**–**C**) Adjacent sections from a GemOE tumor IHC stained for geminin (A), c-Abl (B) and HMGB1 (C). (**D**) The level of circulating HMGB1 measured using specific ELISA assay performed on serum isolated from samples collected 7 weeks after mice were injected in mammary fat pads with naïve HME cells (*n* = 10, no tumors developed) or GemOE cells (*n* = 30, tumor-bearing, *p = 0.00042*). Two different sets (**E, G, I** and **K**) and (**F, H, J** and **L**) of adjacent sections from GemOE orthotopic mammary tumors stained with H & E (E and F), or IHC stained for geminin (G and H), HMGB1 (I and J) as well as HIF-1α (K) or hypoxyprobe (L). N denotes necrosis within these tumors that are shown adjacent to the hypoxic cells as indicated by high HIF-1α or hypoxyprobe staining. These cells are also expressing cytoplasmic HMGB1. (**M**) The levels of HMGB1 detected using specific ELISA assay released from iGem9, iG197, iG240 or iG257 cells grown under normoxic (**N**) or hypoxic (H) conditions. Experiments were done in triplicates 3 different times, **represents *p < 0.001.* (N) The level of acetylated HMGB1 passively diffused from naïve HME, iG197, iG240 or iG257 cells after repeated freeze and thaw cycles.

To re-enforce the data shown above in a controlled system of GemOE tumors, IHC staining of sections from GemOE orthotopic tumors (*n* = 30) for geminin, c-Abl and HMGB1 was done. Like human tissues, GemOE tumors sections (Figure 4A) showed exclusive nuclear c-Abl (Figure 4B), and cytoplasmic HMGB1 (Figure 4C) expression. Moreover, serum isolated from peripheral blood collected at the time of euthanasia of these mice that developed tumors following injection of iGemOE cells (*n* = 30) and from mice injected with naïve HME cells that developed no tumors (*n* = 10) were processed for HMGB1 specific ELISA. While mice injected with naïve HME cells contained 16.05 ng/ml of HMGB1 in the circulation, GemOE-tumor bearing mice contained 168.5 ng/ml of HMGB1 in their circulation (*p = 0.00042*, Figure 4D), thus supporting the above conclusion that GemOE phenotype is associated with cytoplasmic expression and/or extracellular secretion of HMGB1, *in vivo*.

Additionally, adjacent sections from these orthotopic mammary GemOE tumors were stained with anti-geminin, -HMGB1, -HIF-1α antibodies or for hypoxyprobe-1. Due to their fast growing nature, GemOE tumors contain large necrotic cores that were easily detected in H & E sections (see N in Figure 4E and 4F). Surrounding these necrotic cores, surviving geminin-overexpressing cells (Figure 4G and 4H) that express cytoplasmic HMGB1 (Figure 4I and 4J) are HIF-1α^+^ (Figure 4K) or hypoxyprobe^+^ (Figure 4L) hypoxic cells.

iG197, iG240 and iG257 cells were grown under normoxic (N) or hypoxic (H) conditions for 24h. HMGB1 specific ELISA showed that compared to iG197, iG240 and iG257 growing under N condition these cell lines growing under H condition secrete ∼3 fold higher HMGB1 (Figure 4M). These data establish the active secretion of Ac-HMGB1 from GemOE cells *in vitro* and surviving GemOE tumor cells in tumor cores *in vivo*, which is exacerbated by hypoxic environment within the core.

To determine whether the passively diffused HMGB1 from necrotic GemOE tumor cells within the core *in vivo* is also acetylated, naïve HME, iG197, iG240, and iG257 cells were exposed to several rounds of freeze-thaw cycles to mimic the *in vivo* necrotic conditions. Passively diffused proteins were then IPd using an anti-HMGB1 antibody, which showed almost equal amounts of HMGB1 in naïve HME and GemOE tumor cell lines (Figure 4N, upper panel). Stripping and re-probing the membrane with an anti-acetyl-lysine antibody showed that while no Ac-HMGB1 was detected in necrotic naïve HME cells, high level of Ac-HMGB1 was detected in the necrotic GemOE tumor cells (Figure 4N, lower panel). Taken together, these data suggest that hypoxia exacerbates secretion of HMGB1 from GemOE cells, *in vitro* and *in vivo* and that whether actively secreted from surviving/hypoxic or passively diffused from dying/necrotic GemOE cells *in vitro* or GemOE cells within tumor cores *in vivo* HMGB1 is acetylated.

### MSCs entrained by GemOE tumor cells express RAGE and CXCR4

**Figure 5:**
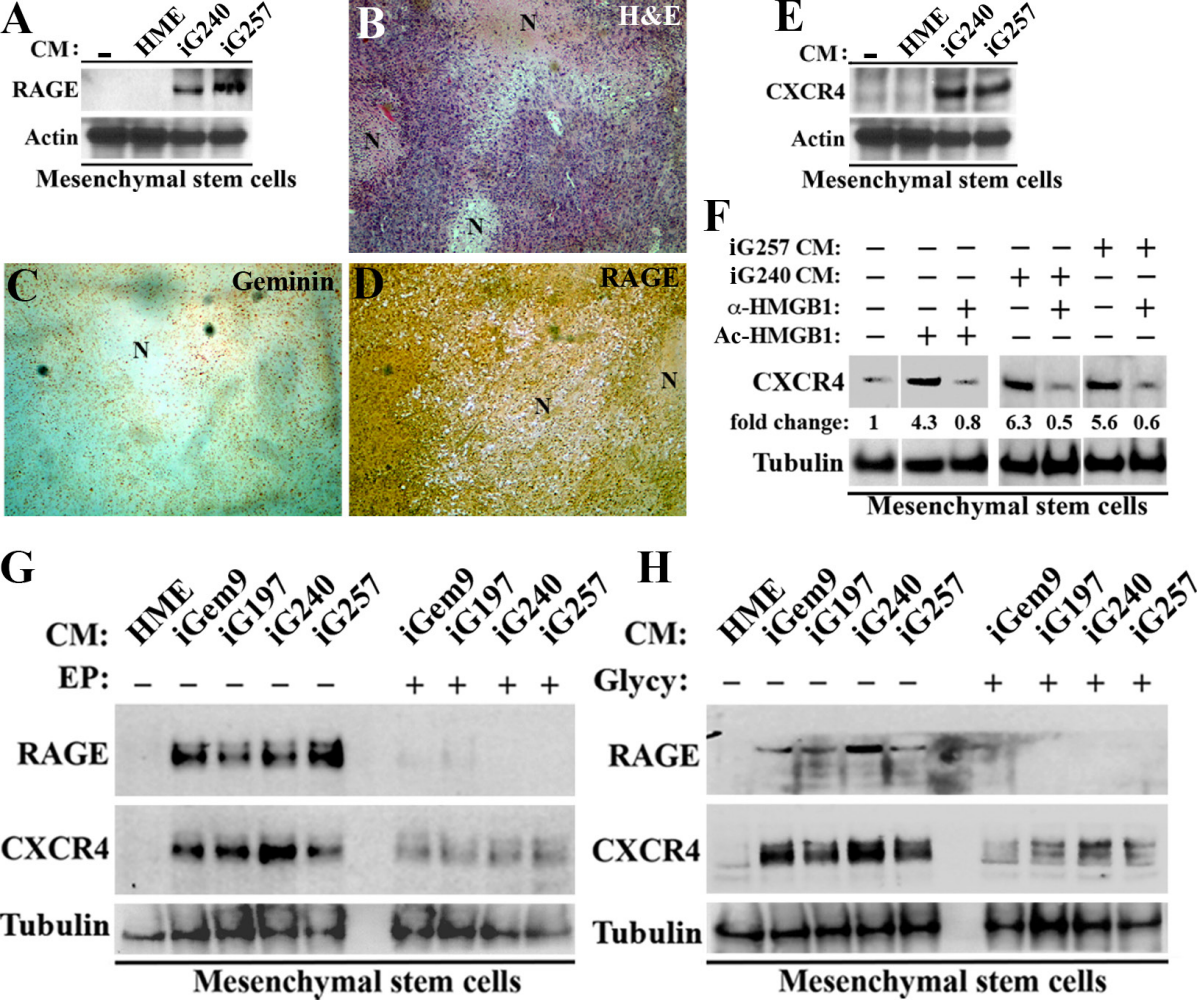
GemOE tumor cells induce MSCs to express RAGE and CXCR4 (**A**) The levels of RAGE on the surface of early passage MSCs following exposure to none (−), naïve HME, iG240 or iG257 CM for 24 h. (**B**–**C**) adjacent sections from GemOE orthotopic mammary tumor stained with H & E (B) or immunohistochemically stained with geminin (C) or RAGE (**D**). Note that as oppose to geminin staining that is detected in mammary cells only, RAGE staining is detected in mammary cells as well as stromal cells recruited into these tumors *in vivo*. (**E**) The levels of CXCR4 on the surface of early passage MSCs following exposure to none (−), naïve HME, iG240 or iG257 CM for 24 h. (**F**) The levels of CXCR4 on the surface of early passage MSCs following exposure to none (lane 1), 10 μg/ml Ac-rHMGB1 (lane 2), 10 μg/ml Ac-rHMGB1 + 10 μg/ml HMGB1 NeuAb (lane 3), iG240 CM alone (lane 4) or + 10 μg/ml HMGB1 NeuAb (lane 5), iG257 CM alone (lane 6) or + 10 μg/ ml HMGB1 NeuAb (lane 7) for 24 h. (**G**) The levels of RAGE and CXCR4 on the surface of early passage MSCs following exposure to naïve HME, iGem9, iG240 or iG257 CM for 24 h in the absence (−) or presence (+) of 200 μM EP. (**H**) The levels of RAGE and CXCR4 on the surface of early passage MSCs following exposure to naïve HME, iGem9, iG240 or iG257 CM for 24 h in the absence (−) or presence (+) of 200 μM Glycyrrhizin. In all parts of the figure experiments were done between 2–3 separate times.

Extracellular HMGB1 is involved among other functions in triggering trafficking of human MSCs into tumors [38, 39]. To understand the mechanism involved, we exposed early passage MSCs to none (grown in the presence of fresh HME medium) or naïve HME, iG240 or iG257 cells CM. Twenty-four hour later membrane proteins were isolated from all cultures and analyzed for known HMGB1 receptors. As oppose to TLR4 that was expressed at high level on the surface of MSCs, naïve MSCs are RAGE-negative (see [−] in Figure 5A). Exposure to naïve HME CM did not enhance RAGE expression on MSCs surface (Figure 5A). In contrast, exposure to iG240 and iG257 cells CM induced high level RAGE on MSCs surface (Figure 5A).

To corroborate these data further, adjacent sections from GemOE-induced tumors were IHC stained with anti-geminin and anti-RAGE antibodies. Again around necrotic cores (see N in H & E stained section, Figure 5B), tumor but not stromal cells expressed geminin (Figure 5C), whereas tumor (see also Figure 2E) and stromal cells (including MSCs) expressed high levels of RAGE (Figure 5D). Together show constitutive RAGE expression on the surface of GemOE tumor cells, but inducible expression on the surface of MSCs through interaction with GemOE cells (cf. Figure 5A).

CXCR4 is a major receptor involved in MSCs recruitment into tumors (see introduction). MSCs membrane proteins from Figure 5A were blotted for CXCR4 expression. Like RAGE, naïve MSCs are CXCR4-negative (or express very low level on their surface, see [−] in Figure 5E) and exposure to naïve HME cells CM caused no change in the level of CXCR4 on the surface of MSCs (Figure 5E). In contrast, exposure to iG240 or iG257 cells CM caused significant increase in CXCR4 level on MSCs surface (Figure 5E). Taken together, these data suggest that a factor secreted by GemOE tumor cells (such as Ac-HMGB1, see below) stimulates expression of RAGE and CXCR4 on the surface of the non-expressing MSCs.

### Ac-HMGB1-RAGE signaling stimulates CXCR4 expression on naïve MSCs surface

To directly assess the role of Ac-HMGB1 secreted from GemOE tumor cells on induction of CXCR4 on the surface of MSCs, we again exposed early passage MSCs for 24 h to fresh HME medium supplemented or not with recombinant HMGB1 (rHMGB1, 10 μg/ml) that was *in vitro* acetylated. Some cultures were exposed to Ac-rHMGB1 plus anti-HMGB1 neutralizing antibody (HMGB1 NeuAb, 10 μg/ml). Unlike naïve MSCs that showed low/no detectable level of CXCR4 on their surface (taken as 1, 1st lane in Figure 5F), MSCs exposed to Ac-rHMGB1 showed > 4fold increase in the expression of CXCR4 on their surface (compare 2nd to 1st lane in Figure 5F). HMGB1 NeuAb completely blocked the induction of CXCR4 by Ac-rHMGB1 (compare 3rd to 2nd lane in Figure 5F).

Additionally, we exposed early passage MSCs for 24 h to iG240 or iG257 cells CM that was supplemented or not with 10 μg/ml HMGB1 NeuAb. Here too compared to naïve MSCs grown in fresh HME medium supplemented, MSCs exposed to iG240 cells CM (compare 4th to 1st lane in Figure 5F) or iG257 cells CM (compare 6th to 1st lane in Figure 5F) showed 5-7fold increase in CXCR4 level on their surface. This induction was also completely blocked by the HMGB1 NeuAb (compare 5th to 4th lane and 8th to 7th lane, respectively in Figure 5F). Together show that whether diffused out of necrotic GemOE cells or released from hypoxic GemOE cells within tumor cores, Ac-HMGB1 upregulates CXCR4 on the surface of MSCs most likely through activation of RAGE.

To directly explore this possibility, early passage MSCs were grown in naïve HME CM for 24 h, in the presence of CM from iGem9, iG197, iG240 or iG257 cells pre-treated with vehicle or 200 μM EP [30] for 24 h or CM from the same cell lines supplemented or not with 200 μM glycyrrhizin (Glycy, inhibits extracellular HMGB1 binding to RAGE) [40]. Twenty-four hours later membrane proteins from all cultures were collected and RAGE and CXCR4 levels in them were examined. Again, in the presence of naïve HME cells CM MSCs showed no RAGE or CXCR4 expression on their surface (see 1st lanes in Figure 5G and 5H). In contrast, in the absence of the drugs iGem9, iG197, iG240 or iG257 cells CM induced expression of RAGE and CXCR4 on the surface of MSCs (see [−] in Figure 5G and 5H). These inductions were almost completely blocked when MSCs were exposed to CM collected from iGem9, iG197, iG240 or iG257 cells pre-exposed to EP (see under [+] in Figure 5G) or Glycy (added at the time CM was added to MSCs, see under [+] in Figure 5H). Together re-enforces the aforementioned conclusion that induction in CXCR4 expression on the surface of naïve MSCs is directly controlled by Ac-HMGB1 activation of RAGE *in vitro* and *in vivo* and suggest that GemOE cells through secreting Ac-HMGB1 recruit MSCs to their vicinity in tumor cores.

### GemOE tumor cells recruit MSCs to their vicinity using CXCR4 signaling, *in vitro*

**Figure 6:**
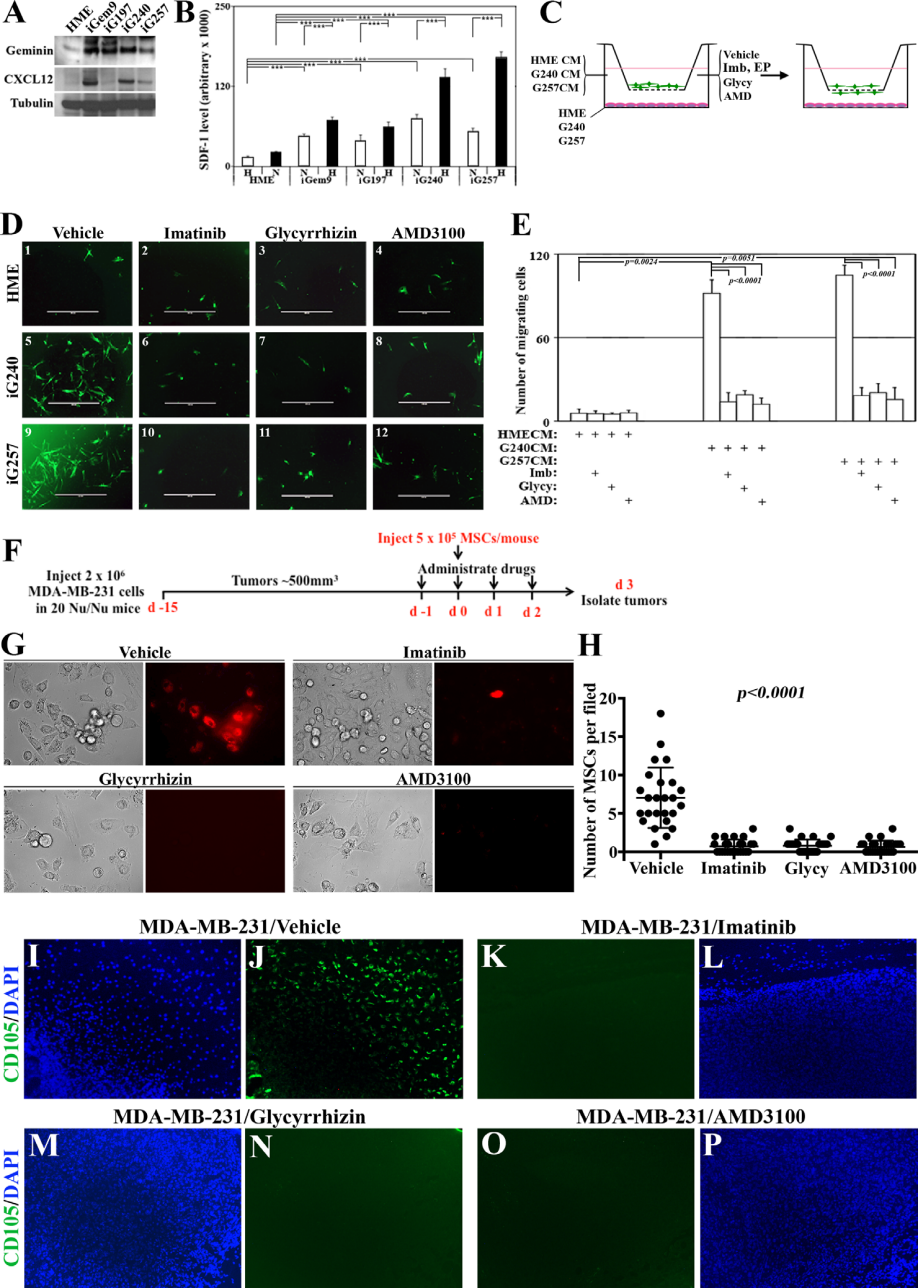
Novel signaling circuit involved in recruitment of MSCs to the vicinity of GemOE tumors, *in vitro* and *in vivo* (**A**) The levels of geminin and SDF1/CXCL12 expressed in naïve HME, iGem9, iG197, iG240 or iG257 cells. (**B**) The levels of SDF1/CXCL12 secreted from naïve HME, iGem9, iG197, iG240 or iG257 cells grown under normoxic (N) or hypoxic (H) conditions as detected using specific ELISA assay. Experiments were done in triplicates 3 different times, ***represents *p < 0.0001.* (**C**) Schematic representation showing the experimental strategy used in (D and E). (**D**) Representative images showing the recruitment of GFP-MSC to the lower side of Boyden chambers inserts when naïve HME, iG240 or iG257 cells were layered on the lower wells and cells were grown in the presence of vehicle (1, 5 and 9, respectively), 10 μM imatinib (2, 6 and 10, respectively), 200 μM Glycyrrhizin (3, 7 and 11, respectively) or 10 μM AMD3100 (4, 8 and 12, respectively). (**E**) Quantitative analysis of the data represented in (D). Experiments were done in triplicates 3 different times, **represents *p < 0.001* and ***represents *p < 0.0001*. (**F**) Schematic representation of the *in vivo* strategy used in the experiments used in G-P. (**G**) Representative images of cell lines generated from subcutaneously generated MDA-MB-231 tumors in Nu/Nu mice that were intracardiac injected with red-labeled MSCs and treated with vehicle, imatinib, glycyrrhizin or AMD3100 for 4 days (day −1, 0, 1 and 2 with regards to MSCs injection) collected 24 h after the last drugs administration (i.e. day 3 after MSCs injection). (**H**) Quantitative analysis of the data represented in (F). (**I**–**P**) Fluorescence immunohistochemical staining using anti-human CD105 (human MSC marker, green) and counterstained with DAPI (blue) of sections from the MDA-MB-231 tumors described in F. Except the *in vivo* part, all parts are of experiments that were done 2–3 separate times.

To directly assess this hypothesis we analyzed the level of CXCR4 ligand SDF1 expression within or secreted from iGemOE cells. According to Western blot analysis comparing naïve HME cells to iGem9, iG197, iG240 or iG257 cells showed that tumor cells contain much higher levels of SDF1 (Figure 6A). Additionally, specific SDF1 ELISA showed that under normoxic conditions naïve HME cells secrete significantly lower level of SDF1 compared to iGem9, iGem197, iGem240 and iGem257 (white bars in Figure 6B). Together suggest that production and secretion of SDF1 is elevated in GemOE tumor cells. Secretion of SDF1 by iGem9, iG197, iG240 and iG257 cells and not by naïve HME cells was further stimulated under hypoxic conditions (for 24 h, black bars in Figure 6B). We concluded that hypoxia also exacerbates the production and secretion of SDF1 from GemOE cells and could be involved in enhancing MSCs recruitment to the vicinity of GemOE cells in tumor cores.

To experimentally explore this, naïve HME, iG240 and iG257 cells were plated in the lower well of 8 μm pores Boyden chambers. Green fluorescence protein (GFP)-expressing MSCs were placed in the inserts and vehicle, imatinib (10 μM), EP (200 μM), Glycy (200 μM) or AMD3100 (a specific CXCR4 inhibitor, 10 μM) were added to CM (Figure 6C, left). Twenty-four hours later, GFP-MSCs migrated to the bottom of the inserts were counted and photographed (Figure 6C, right). In vehicle-treated cultures, naïve HME cells recruited ∼5 ± 1 GFP-MSCs/high magnification field (HF) to their vicinity (Figure 6D_1_ and 6E). In contrast, vehicle-treated iG240 cells recruited ∼102 ± 11 MSCs/HF (Figure 6D_5_ and 6E) and iG257 recruited ∼111 ± 8 MSCs/HF (Figure 6D_9_, and 6E). Recruitment by naïve HME cells was not affected when cells were treated with imatinib (Figure 6D_2_ and 6E), EP (not shown), Glycy (Figure 6D_3_ and 6E) or AMD3100 (Figure 6D_4_ and 6E). On the other hand, significant decrease in the number of GFP-MSCs recruited to the vicinity of iG240 and iG257 cells in the presence of imatinib (Figure 6D_6_ and 6D_10_, respectively), EP (not shown), Glycy (Figure 6D_7_ and 6D_11_, respectively) or AMD3100 (Figure 6D_8_ and 6D_12_, respectively) was detected. We propose that GemOE tumor cells actively recruit MSCs to their vicinity through the signaling circuit “GemOEàHMGB1/RAGEàSDF1/ CXCR4.

### GemOE tumor cells recruit MSCs to their vicinity using CXCR4 signaling, *in vivo*

To define the role of this signaling circuit in recruiting MSCs to breast tumors *in vivo*, 2 × 10^6^ cells of the TNBC cell line; MDA-MB-231 was subcutaneously injected in the back in 20 Nu/Nu mice (Figure 6F). When tumors reached ∼500 mm^3^ mice were divided into 4 groups (5 mice each) that received on day −1 vehicle, 40 mg/kg imatinib, 200 mg/kg Glycy or 3.5 mg/kg AMD3100 (Figure 6F). The next day all mice were intracardially injected with red-labeled MSCs (Red-MSCs, 5 × 10^5^ cells/mouse, Figure 6F). Each group of mice was then treated with the above drugs on the day of MSCs injection (i.e. day 0, Figure 6F), followed by 2 more injections of the aforementioned drugs on day 1 and day 2 following MSCs injection (Figure 6F). Mice were left for another day before tumors were collected (Figure 6F).

First, we used these tumors to generate cultures that were analyzed 48 h later under fluorescence microscope for the presence of Red-MSCs. A large number of Red-MSCs was found within cultures generated from vehicle treated mice (see Figure 6G and 6H), indicating enhanced MSCs recruitment ability, *in vivo*. In contrast, cultures generated from tumors treated with imatinib, Glycy or AMD3100 contained very few, if any, Red-MSCs within them (Figure 6G and 6H).

Second, paraffin-embedded sections generated from these tumors were IHC stained with an anti-human specific MSCs marker; CD105. We again found that vehicle-treated tumors contained high number of CD105^+^ cells (Figure 6I and 6J) indicating enhanced MSCs recruitment ability, *in vivo*. In contrast, mice treated with imatinib (Figure 6K and 6L), Glycy (Figure 6M and 6N) or AMD3100 (Figure 6O and 6P) showed no CD105^+^ staining indicating significant loss of their ability to attract MSCs, *in vivo*. We propose that similar to *in vitro* data, *in vivo* GemOE cells recruit MSCs to their vicinity through secreting Ac-HMGB1 that promotes expression and activation of RAGE, which in turn induces CXCR4 expression in MSCs that responds to high levels of SDF1 secreted from GemOE tumor cells. Inhibiting geminin activity through inactivating c-Abl, HMGB1 secretion from GemOE cells or activation of RAGE in MSCS or CXCR4 activity in MSCs abrogates the migration of MSCs towards SDF1 expressing GemOE cells.

### MSCs entrained by GemOE enhance aggressiveness in GemOE tumor cells, *in vitro*

**Figure 7:**
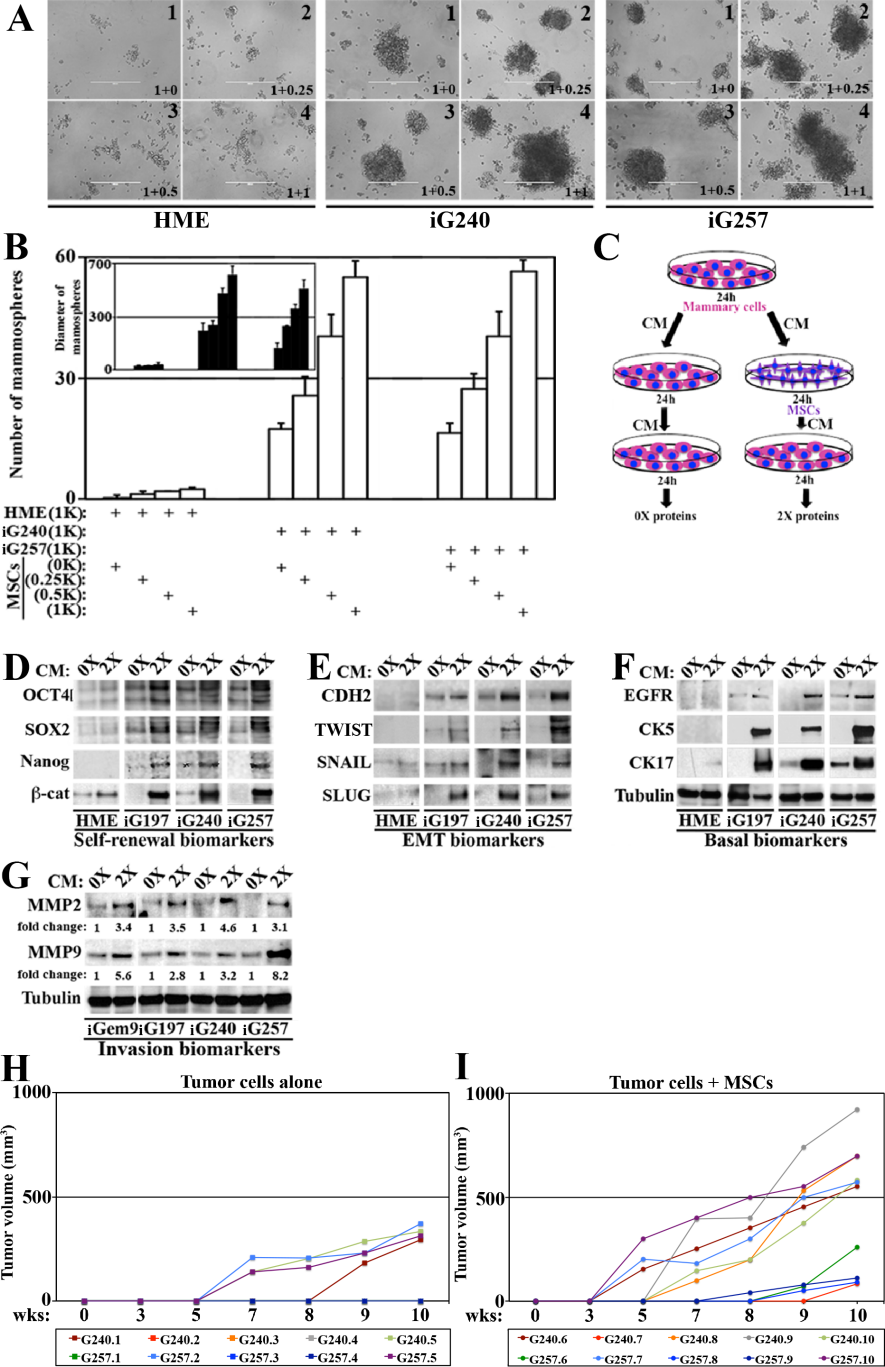
MSCs enhance GemOE aggressiveness, *in vitro* and *in vivo* (**A**) Mammosphere formed when 1000 naïve HME, iG240 or iG257 cells were plated alone or mixed with 250, 500 or 1000 MSCs. (**B**) Quantitative analysis of the data represented in (A). Experiments were done in triplicates 3 different times with *p* values between *0.0010*–*0001*. Inset shows diameter of mammosphere developed in (A). Experiments were done in triplicates 3 different times with *p* values between *0.0010–0001*. (**C**) Schematic representation of the experiments described in (**D**–**F**). The levels of indicated self-renewal biomarkers (D), EMT biomarkers (E) or basal biomarkers (F) in proteins extracted from naïve HME, iG197, iG240 or iG257 cells following the 0× or 2× treatment. (**G**) Similar experiments showing the levels of the invasion proteins MMP2 and MMP9 in proteins extracted from iGem9, iG197, iG240 or iG257 cells following the 0× or 2× treatment. (**H**) The size of orthotopic tumors developed in Nu/Nu mice injected with 2 × 10^6^ iG240 (T1-T5) or 2 × 10^6^ iG257 (T6-T10) cells within 10 weeks set limit for the experiment. (**I**) The size of orthotopic tumors developed in Nu/Nu mice injected with 2 × 10^6^ iG240 + 3 × 10^5^ MSCs (T11–T15) or 2 × 10^6^ iG257 + 3 × 10^5^ MSCs (T16–T20) cells within 10 weeks set limit for the experiment. In all parts of the figure experiments were done between 2–3 separate times.

We next used mammosphere formation assay to measure the impact of MSCs entrained by GemOE on the aggressiveness of breast tumor cells. In low-binding culture dishes, a thousand naïve HME, iG240 or iG257 cells were plated alone or admixed with MSCs at 1:0.25, 1:0.5 or 1:1 ratios (mammary cells:MSCs). Naïve HME cells alone failed to form any mammospheres (Figure 7A_1_ and 7B) and mixing with MSCs even at 1:1 ratio did not increase their mammospheres’ forming ability (see Figure 7A_2-4_ and Figure 7B) or the diameter of the few mammospheres formed (Figure 7B inset). In contrast, iG240 and iG257 cells alone formed many mammospheres (7A_5_ and 7A_9_ and 7B) with relatively large diameter size (Figure 7B inset). Mixing with MSCs at 1:0.25 showed only slight increase in the number of mammospheres developed by iG240 or iG257 cells (Figure 7A_6_ or 7A_10_, respectively) but had no impact on the diameter of these mammospheres (Figure 7B, inset). However, admixed with 1:0.5 or 1:1, MSCs significantly increased the numbers (Figure 7A_7,8_ and 7A_11,12_, respectively and 7B) and the diameter (Figure 7B inset) of the mammospheres formed by iG240 and iG257. Together suggest that once in the vicinity of GemOE tumor cells, MSCs activate stemness/aggressiveness in tumor cells.

To further establish that, media conditioned by naïve HME, iG197, iG240 or iG257 cells for 24 h was added onto the same cell lines or onto MSCs for 24 h before this re-conditioned media were added to cultures of the same cell line (Figure 7C). Proteins isolated from mammary cellsàMSCsàmammary cells were called 0×, whereas proteins from mammary mammary cellsàMSCsàmammary cells were called 2× (Figure 7C). In agreement with the above data, compared to 0 × proteins isolated from naïve HME cells, 0× proteins isolated from iG197, iG240, iG257 contained higher levels of some self-renewal (e.g., OCT4 and SOX2), EMT (e.g., CDH2 and SNAIL) and basal (e.g., EGFR and CK17) (Figure 7D–7F) biomarkers. Impressively, in 2 × proteins isolated from iG197, iG240, iG257 cells the levels of the self-renewal (OCT4, SOX2, NANOG and β-catenin, Figure 7D), EMT (CDH2, TWIST, SNAIL and SLUG, Figure 7E) and basal (EGFR, CK5 and CK17, Figure 7F) biomarkers were significantly increased. The same treatment slightly increased the levels of some, such as β-catenin, SLUG and CK17 in naïve HME cells (Figure 7D–7F).

Moreover, compared to 0× proteins isolated from iGem9, iG197, iG240 and iG257, 2× proteins showed dramatic increase in the levels of the invasion promoting proteins (MMP2 and MMP9, Figure 7G). Indeed, compared to 0 × isolated proteins from iGem9, iG197, iG240 and iG257 each taken as 1, we detected 3-5fold increase in the levels of MMP2 and between 2-8fold increase the levels of MMP9 in 2× proteins isolated from these cell lines (Figure 7G). Taken together, these data re-enforce the aforementioned conclusion that MSCs entrained by GemOE tumor cells reciprocally increase tumor cells aggressiveness *in vitro*.

### GemOE-entrained MSCs promote GemOE-tumor cells aggressiveness, *in vivo*

Finally, to assess the role of MSCs on GemOE aggressiveness *in vivo*, we used orthotopic tumor formation model. Previously, injection of 5 × 10^6^ GemOE cells into SCID or Nu/Nu mice mammary fat pads promoted orthotopic GemOE tumor of ∼1.5 cm^3^ tumors within ∼7 weeks. Therefore, we elected to test tumor formation by 2 × 10^6^ cells from cell lines generated from these tumors (namely G240 and G257) when co-injected or not with MSCs and set 10 weeks as the endpoint. Twenty Nu/Nu mice divided into 4 groups (5 mice each) were injected into the 2nd left mammary fat pads with G240 cells alone (group 1), 2 × 10^6^ G240 cells + 3 × 10^5^ MSCs (group 2), 2 × 10^6^ G257 cells alone (group 3) and 2 × 10^6^ G257 cells + 3 × 10^5^ MSCs (group 4). All mice were kept on Dox-supplemented drinking water and tumor growth was monitored 3 times a week using caliper.

Only 2 mice (T1 and T5) from group 1 and 2 mice from group 3 (T7 and T10) developed tumors (Figure 7H) within the 10 weeks allowed. In contrast, all 5 mice from group 2 and group 4 developed tumors within the 10 weeks (Figure 7I). Moreover, tumors developed in mice in group 1 and 3 showed long latency, i.e. began to appear by 5–8 weeks after injection and were small in size with volumes remaining below 500 mm^3^ by 10 weeks (Figure 7H). In contrast, tumors developed in mice from group 2 and 4 showed short latency, i.e. appeared much earlier and were larger in size with some tumor reaching ∼1000 mm^3^ by 10 weeks (Figure 7I). Indeed, 3 tumors T11 (iG240 + MSCs), T17 and T20 (iG257 + MSCs) began to appear by week 3 and reached ∼750 mm^3^ by week 10 (Figure 7I). Another 3 tumors T13, T14 and T15 (iG240 + MSCs) began to appear by week 5 and grow fast to reach 700-900 mm^3^ by week 10 (Figure 7I). Finally, tumor T12 (iG240 + MSCs) and T16, T17 and T19 (iG257 + MSC) tumors began to appear by week 7–9 and grow relatively slow to reached 100–300 mm^3^ by week 10 (Figure 7I). These data show that even at this low number, GemOE cells are able to form tumors. However, admixed with MSCs GemOE cells show significant increase in the tumor take and number. MSCs also increased the volume of each tumor developed, a reflection of the increase in growth rate. Finally, co-injecting MSCs significantly decreased the latency of tumor formation by ∼2 weeks. Taken together, these data show that even *in vivo* the proximity of MSCs enhances GemOE tumor cell tumorigenic potential.

## DISCUSSION

There has been intensive search for mechanisms that dictate which cells within a given breast tumor become metastatic precursors. Even more intensive search has been for defining whether these mechanisms are intrinsic or induced by the microenvironment. It is well appreciated that hypoxia and necrosis within tumor cores are intimately involved in the development of more aggressive tumor cells [41]. Cells located within these cores are exposed to the harshest conditions within the tumor. If they survive, they most likely become more aggressive. In the current work we expand on this by suggesting that tumor cells within the necrotic/hypoxic GemOE breast tumors core become breast cancer metastasis precursors through recruiting and interacting with MSCs [42].

**Figure 8:**
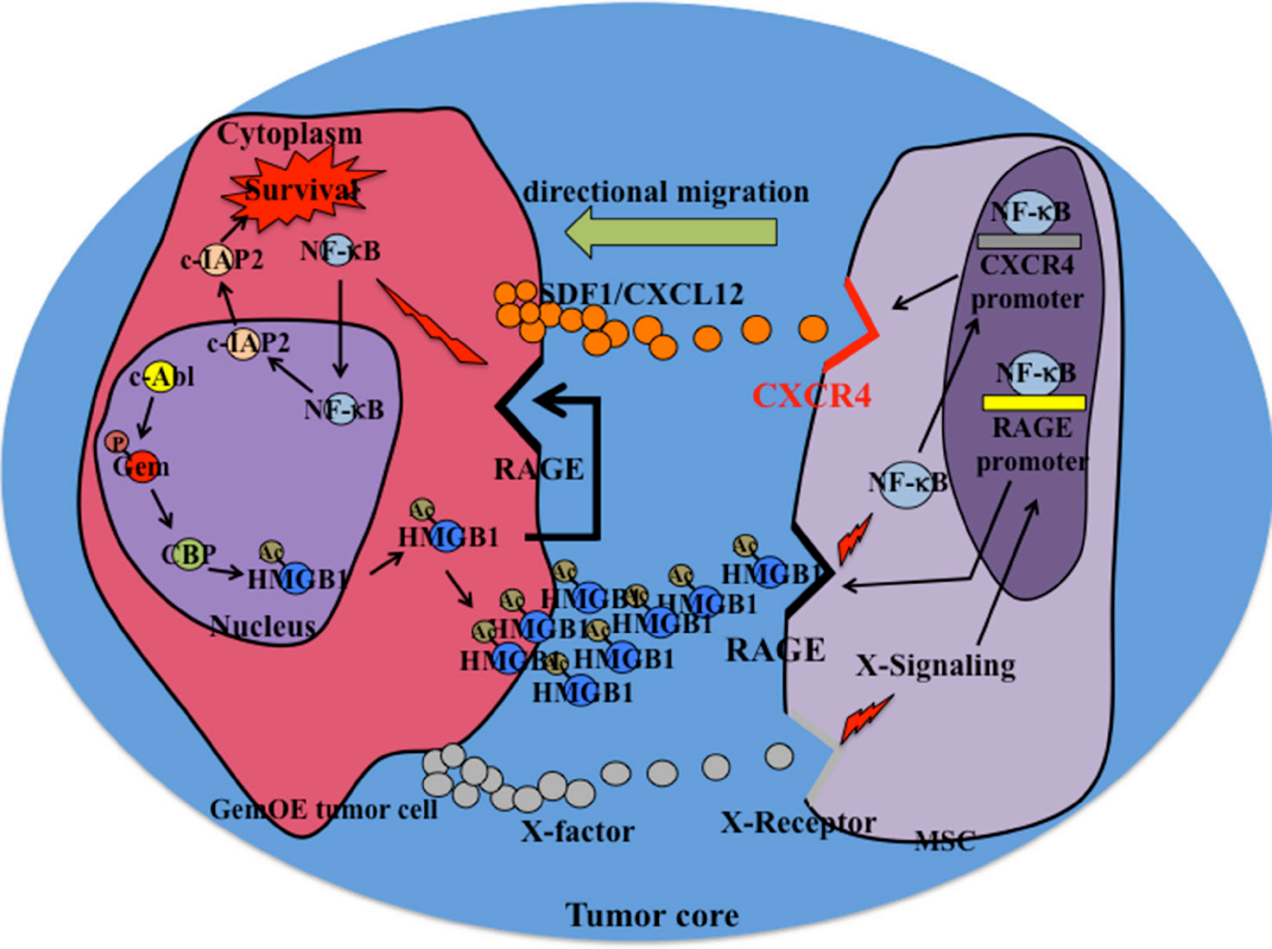
Schematic representation of the data presented in this article

Within this core, dying/necrotic or surviving/hypoxic GemOE tumor cells release Ac-HMGB1. RAGE and not TLR4 is constitutively expressed by all GemOE tumor cells. This suggests that through autocrine/paracrine binding to these RAGE receptors, Ac-HMGB1 activates NF-κB signaling and promotes survival of these hypoxic GemOE tumor cells within tumor cores. In contrast, only after exposure to GemOE tumor cells CM, RAGE expression was initiated on the surface of MSCs. Activation of RAGE expression and activation was also directly correlated with CXCR4 induction and activation on the surface of MSCs also through activating NF-κB signaling [4, 5]. These CXCR4 expressing MSCs later migrate towards SDF1 secreting GemOE cells in the cores. This suggests that within the cores RAGE plays different functions. We also showed that HMGB1 passively diffused out of dying necrotic cells in the cores is also acetylated and thus could also be involved in the directed migration of MSCs towards SDF1 secreting GemOE tumor cells within the cores. Schematic representation of the conclusions from our studies is shown in Figure 8.

How Ac-HMGB1 induces RAGE expression on the surface of RAGE-negative naïve MSCs is currently unknown. However, it is possible that low level of RAGE receptor below our level of detection exists on the surface of MSCs is initially activated by Ac-HMGB1, which promotes further expression of RAGE as an inflammatory response in a NF-κB-dependent manner [19, 29]. Alternatively, other soluble factors in GemOE CM, e.g., IL-6 may initially activate RAGE expression and once it is located on the surface, binding by Ac-HMGB1 maintain high-level expression on the surface. Also possible is that the initial effect of Ac-HMGB1 is through activating TLR4, then switching to RAGE once expressed. Future analysis will focus on 2 major topics were not studied here; direct comparison between tumor areas with and without necrosis with regards to the level of MSCs present, and define whether there are obvious differences in GemOE/TNBC tumor samples with regards to the level of lymphocytes infiltration and amount of MSCs present.

Our data also clearly show that once in the vicinity of GemOE tumor cells within the tumor core, MSCs reciprocally interact with GemOE tumor cells and increase their aggressiveness phenotype. Bi-directional crosstalk between tumor cells and MSCs has been extensively demonstrated in a variety of cancers. Our data shows that MSCs entrained by this crosstalk augment GemOE TIC, EMT and most importantly basal phenotypes [this study and 24, 25]. TICs and EMT enhance tumor progression [43], drug-/radio-resistance and post-treatment relapse [44] in breast cancers [45–47]. It is thus important to identify the cytokines and the intracellular signaling involved in these crosstalk to help the development of more efficient anti-breast cancer therapies.

Intriguingly, IHC analysis performed on sections from the original GemOE tumors used to generate the cell lines, G197, G240 and G257 showed high (although patchy) staining level for several TIC, EMT and basal biomarkers presented here (manuscript in preparation). The fact that their levels dropped in the cell lines (see 0× in Figure 7D–7G) and increased only when tumor cells were exposed to MSCs re-CM medium (see 2× in Figure 7D–7G) suggests that cells differentiated in culture lose expression of these biomarkers. The fact that the expression was reinstated suggests their de-differentiation in culture driven by interaction with MSCs and point out to the enormous plasticity of these GemOE/TNBC tumor cells. In support of that MSCs derived from ovarian cancers were more effective in inducing cancer growth, especially the putative TIC/CSCs phenotype than normal human bone marrow isolated MSCs [48, 49].

Finally, the current studies clearly show an important role for c-Abl, HMGB1 and CBP in enhancing GemOE/TNBC cells aggressiveness. It is not surprising that a CBP (as well as p300 and GCN) small molecule inhibitor was recently shown to inhibit TNBC cell growth *in vitro* and *in vivo* [50]. SIRT1 activating resveratrol could also be a good candidate for treating GemOE/TNBC [15, 16]. Additionally, based on the work presented here, combinatorial therapies targeting geminin through inhibiting c-Abl with imatinib plus CBP inhibitor and/or drugs that activate SIRT1 and/or inhibitors Ac-HMGB1 secretion or binding to RAGE could be pursued as valid therapies to prevent MSCs recruitment and enhancement of GemOE/TNBC tumor aggressiveness.

## MATERIALS AND METHODS

### HME cells generation protocol

Normal mammary epithelial cells isolated in our laboratory from tissues during mammary gland reduction surgeries, or were purchased Clonetics/Cambrex (Waltersville, MD). The isolated cells were named normal HME cells and were infected with a retro-plasmid expressing TERT for immortalization. After selection with appropriate antibiotic, the resultant cells were expanded and frozen for future use at early passages. Although immortalized the karyotype of the cells was tested at every 10 passages and was found to be normal. These cells were named naïve HME cells. Naïve HME cells were infected with a retrovirus carrying a TetOn plasmid (Clontech) selected, expanded and froze. Naïve HME cells expressing TetOn were next infected with a retrovirus expressing an inducible TRE-geminin allele (Clontech). Infected clones expressing geminin after incubation with Doxycycline (2 μg/ml) were chosen for further use. Gem9 is one such clone that express similar amount of geminin as TNBC cells and therefore was chosen to develop orthotopic tumors. For more information regarding these protocols [27].

### HME and Gem9 cells synchronization and transfection protocol

Detail of this protocol was first published in [27]. In brief, HME and Gem9 cells were incubated in growth factor-free medium for 72 hours to synchronize cells in G_0_/G_1_ phase (> 95%) G_0_/G_1_ cells were then released from arrest in medium containing growth factors, and 16 hours (S phase), 22 hours (G_2_/M phase) or 24 hours (M/G_1_ phase) later cells were collected and analyzed. For more information regarding this protocol [27, 51]. Cell transfection with double-stranded RNA interference (siRNA) reagent was performed as described by [51]. In brief, cells were transfected (0 hours) in serum-free medium with a relevant by a standard method using oligofectamine. At 24 hours, the medium was changed, and growth factor-containing MEBM (Clonetics/Cambrex, Waltersville, MD) was added. Small interfering RNA (siRNA) used were siGem: TGAGCTGTCCGCAGGCTTT, scrambled siGem: TGATTTGTCCGCAGCTGGC and c-Abl siRNA was premade from Dharmacon. The silenced luciferase (siLuc) was from previously published data. For more information see [24–27].

### Mesenchymal stem cells (MSCs)

The human MSCs were purchased from Texas A & M HSC COM Institute for Regenerative Medicine who isolated, verified and propagated the cells.

### Cell culture and drug treatment

Breast cancer cell lines were maintained in RPMI medium (Invitrogen) supplemented with 10% FBS and antibiotics. Doxycycline and Glycyrrhizic acid ammonium salt were from Sigma, Imatinib and Ethyl pyruvate (EP) were from Toronto Research Chemicals Inc. (TCR) and AMD3100 octahydrochloride was from TOCRIS.

### Antibodies

A mouse anti-geminin monoclonal antibody was developed and extensively tested in our laboratory [27], 2 different mouse monoclonal anti-c-Abl were used and gave essentially identical results, one was from (Cell Signaling, #2862) and the other from (Santa Cruz, sc-23), rabbit anti-Sp1 (Santa Cruz, sc-14027), rabbit anti-acetylated-lysine (Cell Signaling, #9441), mouse anti-Actin (Calbiochem, cat. # cp01), rabbit anti-CBP (Cell Signaling, #4772], rabbit anti-H2B (abcam, ab18977), mouse anti-CD105 (Abcam, ab114052), two different HMGB1 antibodies were used here; mouse anti-HMGB1 (abcam, ab77302) and rabbit anti-HMGB1 (Millipore, #07-584), rabbit anti-Tubulin (abcam, ab11320), rabbit anti-NF-κB/p65 (Santa Cruz, sc-372), rabbit anti-IκB (Santa Cruz, sc-371), rabbit anti-c-IAP2 (Abnova, PAB0253), rabbit monoclonal anti-RAGE (abcam, ab172473), mouse anti-HIF-1α (Novus, NB100-105), rabbit anti-CXCR4 (abcam, ab2074), rabbit anti-SDF1 (Cell Signaling, #3740), mouse anti-OCT3/4 (Santa Cruz, sc-5279), mouse anti-Sox2 (Cell Singling, #4900), goat anti-Nanog (R & D, AF1997), rabbit monoclonal anti-Slug (Cell Signaling, #9585), mouse anti-Twist (abcam, ab50887), rabbit anti-Snail (abcam, ab82846), mouse anti-N-Cadherin (BD, 610920), rabbit monoclonal anti-EGFR (Cell Signaling, #4267), rabbit monoclonal anti-CK5 (abcam, ab75869), rabbit monoclonal anti-CK17 (abcam, ab51056), goat anti-MMP2 (Santa Cruz, sc-6838), goat anti-MMP9 (Santa Cruz, sc-6840). All antibodies were tested and protocols for use on cell culture and on TMA were described in details previously [24–27].

### Chromatin, nuclear and cytoplasmic extracts purification, followed by western blot or immunoprecipitation

The protocols used by [27, 51] were used to isolate total extracts by sonication and chromatin preparations. Briefly, cells at about 75% confluence were washed several times with PBS and trypsinized. After washing cells were resuspended in Buffer A (110 mM KC_2_H_3_O_2_, 15 mM NaC_2_H3O_2_, 2 mM MgC_2_H_3_O_2_, 0.5 mM ethylene glycol tetraacetic acid and 20 mM 4-(2-hydroxyethyl)-1-piperazineethanesulfonic acid (HEPES), pH 7.3). Next, we added 2 mM DTT and 50 μg/ml of digotinin to the cell suspension. The cells were agitated at 4°C for 10 minutes. Nuclei were pelleted by centrifugation in a swinging bucket rotor at 1,500 × g for 10 minutes. They were resuspended in hypotonic Buffer B (1mM HEPES, pH 7.5, and 0.5 mM ethylenediaminetetraacetic acid (EDTA) supplemented with 0.5% NP-40). The nuclear suspension was then agitated at 4°C for 15 minutes and layered on top of a 10 ml sucrose cushion (100 mM sucrose, 0.5 mM Tris HCl, pH 8.5), then centrifuged at 3,500 × g for 15 minutes at 4°C. The chromatin pallet was suspended in 0.25 mM EDTA, pH 8.0, and sonicated three times for 10 seconds each using a Fisher Scientific Model 100 Sonic Dimembrator (Fisher Scientific, Pittsburgh, PA, USA). After sonication, the chromatin suspension was centrifuged twice at high speed for 10 minutes at 4°C, and the supernatants were retained. This chromatin extract was first precleared by agitation for 2 hours at 4°C in the presence of 50 μg of protein A/G Sepharose beads, followed by pelleting of the beads. The supernatant protein concentration was measured, and 500–1000 μg of chromatin protein were routinely immunoprecipitated using 1 or 2 μg of Ab and 50 μl of protein A/G Sepharose beads in a total volume of 1ml of NETN buffer (in which the NaCl concentration was preset at 250 to 500 mM). To isolate nuclear vs. cytoplasmic from the same cell, cells were washed with ice cold PBS, re-suspend in buffer 1 (containing: 10 mM HEPES, 10 mM KCl, 0.5 mM DTT, 1% NP-40) and incubated 10 min at 4°C with gentle agitation, followed by centrifugation for 2 min at max speed and the supernatant was saved as cytoplasmic extract. Nuclear pellet was re-suspended in buffer 2 (containing: 20 mM HEPES, 20% Glycerol, 500 mM KCl, 0.2 mM EPTA, 0.5 mM PMSF, 0.5 mM DTT, 1.5 mM MgCl_2_); incubated 15 min at 4°C with gentle agitation, sonicated and then spun down at max speed to remove non-soluble faction and supernatant was saved as nuclear extract. In all western blot analysis, 25 μg of protein from each extract is loaded on gels.

### Immunofluorescence analysis

Cells were seeded on slide chambers (Lab Tek) at 25% confluence 24 hours prior to processing. Cells were fixed with 4% paraformaldehyde for 10 minutes at room temperature (RT), permeabilized in Triton X-100 buffer (0.5% Triton X-100 in 20 mM HEPES, pH 7.4, 50 mM NaCl, 3 mM Mg_2_Cl, 300 mM sucrose containing 0.5% BSA) for 10 minutes at 4°C. Cells were then incubated for 30 minutes with 5% normal mouse or rabbit serum (MS or RS) in PBS and then incubated for 30 minutes at 37°C with primary antibody. Cells were then incubated with appropriate FITC- or rhodamine-conjugated secondary antibody diluted 1:5000 in 5% MS or RS in PBS for 30 minutes at 37°C. Cover slips were mounted in anti-FADE solution (Vector) supplemented with DAPI.

### *In vitro* acetylation assay

The assay was performed in HAT buffer containing: 50 mM Tris-HCl pH 8.0, 10% glycerol v/v, 1 mM DTT, 0.1 mM EDTA, 0.1 mM PMSF and 10 mM sodium butyrate), with 0.1–0.2 mg/ml rHMGB1 (Sino Biological Inc.), and ∼20 ng/ml GST-CBP fusion proteins. Reactions were incubated for 20 min at 37°C and cooled at 4°C for 10 min before beads and substrate were separated by centrifugation at 1000 *g* for 5 min.

### NF-κB luciferase reporter luciferase assay

The pGL4.32[luc2P/NF-κB-RE/Hygro] vector (Promega, Madison, WI) contains five copies of an NF-κB response element (NF-κB-RE) that drives transcription of the luciferase reporter gene luc2P. GemOE cell lines were transfected with this vector. The next day, cells were treated or not with sRAGE (10 μg/ml, PROSPEC) for 48 h, then luciferase assay (Promega) was used to measure the NF-κB reporter activity. Data were first normalized against Renilla-Luciferase activity.

### Tissue samples and immunohistochemical (IHC) analysis of paraffin-embedded tumor samples

A University of Hawaii IRB committee approved the use of human tumor sample. A training cohort was a commercial TMA (Biomax.us, *n* = 511 samples) containing normal/cancer adjacent tissues (*n* = 66), ductal carcinoma *in situ* (DCIS, *n* = 180), invasive (*n* = 100), and metastatic (*n* = 165) breast tumor samples and a confirmation cohort, consisted of disease-free adult tissues (including; kidney, liver, placenta, spleen and mammary tissues) and a conformational cohort (*n* = 326, breast tumor samples, different stages) acquired from the Hawaiian *Surveillance, Epidemiology and End Results* (SEER) collection constructed in quadruplicate, each containing one sample from a different region of a tumor at 4 μm were used.

### IHC staining scoring

Immunostained slides were scored using a modified protocol of the one described previously [52, 53]. In brief, under light microscope stained sections were scored by counting positive cells in at least 10 high power fields of each tumor section. Score was estimated as follow: 0 = no staining (< 1% of the cells stained); 1 = weak (1–10% of the cells stained); 2 = medium (10–50% of the cells stained); 3 = strong (> 50% of the cells stained). An intensity score was also assigned to each tumor in which the average intensity of positive tumor cells is represented as 0 = none, 1 = weak, 2 = intermediate, and 3 = strong. The positivity and intensity scores were then added to obtain a total score, which ranged from 0 to 6. A pathologist scored slides blindly.

### Overall survival (OS) and repalce-free survival (RFS) and metastasis-free survival (MFS) analysis

Data source for disease-free survival used was the GOBO bioinformatics resource. The association of geminin alone or combined with HMGB1 was investigated for stratified patient cohorts using overall survival (OS) and relapse-free survival (RFS) or metastasis-free survival (MFS) using the PROGgeneV2 - Pan Cancer Prognostics Database (http://watson.compbio.iupui.edu/chirayu/proggene/database). Furthermore, the Kaplan-Meier survival analysis was determined for the gene set in 7 subgroups for a total of 2078 cases with RFS, MFS or OS follow-up from the GEO study as fellows: TCGA, sample size: 593, GSE1456, samle size: 158, GSE2034, sample size: 286, GSE4922, sample size: 248, GSE11121, sample size: 199. Data presented as median cut-off and shown as high expressers vs. low expressers patients for geminin alone or geminin + HMGB1.

### *In vivo* tumorigenicity assay

The University of Hawaii or the University of Mississippi Medical Center IACUC committees approved all animal experiments. Six- to eight-week-old anaesthetized immune-compromised Nu/Nu (Harlan) mice were injected with 2 × 10^6^ cells re-suspended in 100 μl of HME medium or RPMI medium and matrigel (1:1) using a 27-gauge needle either subcutaneously on the back or orthotopically in the 2nd left mammary gland. Tumor initiation was defined as the time when tumors were 3mm in diameter. Mice were sacrificed when the tumors reached ∼1.5 cm^3^ in volume or at 10 wks. Tumor volume was calculated with the formula 4/3πr^3^ (where r is the tumor radius). At the end of the experiments, mice were euthanized by compressed 100% CO_2_ gas, tumors were dissected out, weighed, fixed in formalin, and later cut at 4 μm for histological and immunohistochemical analysis. In some instance, from anesthetized mice (using a mix of oxygen and isoflurane gas), 200 μl of blood was drawn from the heart before they were euthanized.

### Preparation and injection of red-labeled MSCs

To generate red-labeled MSCs we used the PKH26 Red Fluorescent Cell Linker Kit for General Cell Membrane Labeling (Sigma-Aldrich) as per manufacturer procedures. In brief, in RT a 2 × cell suspension was prepared by re-suspending pre-washed 2 × 10^7^ MSCs in serum free medium in 1 ml of Diluent C. A 2 × dye solution prepared by adding 4 μl of PKH26 ethanolic dye solution to 1 ml of Diluent C and mixed well. Rapidly add the 1ml of 2 × cell suspension to 1 ml of 2 × dye solution and immediately mix the sample by pipetting. Leave for < 5 min and stop the reaction by adding 2 ml of serum and incubate 1 min to allow binding of excess dye. Centrifuge cells at 400 × g for 10 min followed by washing 3 times with 10 ml complete medium to remove any unbound dye. Red-labeled cells were injected in mice circulation by injection through the left cardiac ventricle.

### *In vivo* measurement and imaging of orthotopic or subcutaneous tumors

Tumor formation was analyzed with IVIS luciferase machine (Xenogen) weekly and tumor size was measured every 3rd day by caliper (Life Sciences instruments). To analyze tumor formation using the *in vivo* system, mice were i.p. injected using 27-gauge needle with 100 μl of D-luciferin solution (Xenogen) prepared at 15 mg/mL in PBS. Mice were then anesthetized using a mix of oxygen and isoflurane gas. Anesthetized animals were maintained sleep during the imaging procedures by placing the animal nose in a nose cone with a flow of anesthesia gas and take a picture of the tumors.

### *In vivo* drug treatments

MDA-MB-231 cells were injected subcutaneously on the back of SCID mice as described above. Mice with tumors ∼0.5 cm^3^ were injected through the heart with 1 × 10^6^ Red-labeled MSCs and treated with 50 mg/kg/day imatinib, 200 mg/kg/day glycyrrhizin and 3.5 mg/kg AMD3100 by gavage or i.p. injection on day-1, 0, 1 and 2 with regard to MSCs injection. At the end of the experiments tumors were dissected out, weighed and fixed in formalin, cut at 4 μm for histological and immunohistochemical analysis.

### Hypoxia treatment and hypoxyprobe assay and analysis

To perform hypoxia *in vitro*, culture dishes were placed in incubator with 1% O_2_, whereas normoxic conditions were plating cells under commonly used 20% O_2_ incubator. For hypoxyprobe analysis, mice to be analyzed for hypoxia were i.p. injected with Hypoxyprobe^™^-1 (hpi, Burlington, MA) solution (60 mg/kg body weight) dissolved in saline, 60–90 min before mice are sacrificed. IHC of pimonidazole adducts in formalin-fixed, paraffin-embedded tissues was performed using manufacturer's procedures and in [54].

### Isolation of serum and plasma from mouse blood samples

Blood samples collected were divided into 2 fractions. The first was used to isolate serum and the second to isolate plasma. For serum preparation, whole blood was collected in a covered test tube and allowed to clot by leaving undisturbed at RT for ∼30 min. Clot was then removed by centrifuging at 1,000–2,000 g for 10 min at 4°C. The isolated serum is immediately transferred into a clean polypropylene tube aliquoted into 0.5 μl aliquots and stored in −80°C. For plasma preparation, blood was collected into anticoagulant-treated tubes. Cells are removed from plasma by centrifugation for 10min at 1,000–2,000 g at 4°C. The resulting plasma is transferred into a clean polypropylene tube, aliquoted into 0.5 μl aliquots and stored at −80°C. We elected to use serum samples because they are more representative of inflammation.

### ELISA analysis of culture medium or blood serum

Conditioned medium (CM) or serum (Ser) and dilution thereof were generated in PBS. Wells of a PVC microtiter plate were coat with antigen by pipetting 50 μl of the CM or Ser and dilution in triplicates. Plate was covered and incubated for overnight at 4°C. Plate was washed 3 times with PBS. Blocking of none-specific sites was done using blocking buffer (5% BSA in PBS) and the plate was incubated for > 2 h at room temperature followed by washing as above. Diluted primary antibody (determined using pilot experiments) was added to each well and incubated for 2 hours at room temperature followed by washing steps with PBS. Horse-radish peroxidase (HRP) conjugated secondary antibody diluted in blocking buffer and incubated for 1 hour at room temperature washed with PBS. Detection was done using the OPD (o-phenylenediamine dihydrochloride) tables and the end product was measured at 492 nm. All ELISA experiments were done in triplicates at least 3 separate times. *P*-value are * ≤ 0.05, ** ≤ 0.01 and *** ≤ 0.001.

### Statistical analysis

Comparisons of treatment outcomes were tested for statistical differences using the Student *t*-test for paired data. The association of mRNA transcript expression with various clinico-pathologic parameters was also analyzed. Statistical significance was assumed at a *P*-value are * ≤ 0.05, ** ≤ 0.01 and *** ≤ 0.001.

## SUPPLEMENTARY MATERIALS FIGURES


