# Supplementary material for: Geminin overexpression-dependent recruitment and crosstalk with mesenchymal stem cells enhance aggressiveness in triple negative breast cancers
Source: Oncotarget. 2016 Mar 10;7(15):20869–89. doi: 10.18632/oncotarget.8029 (PMC4991498; doi:10.18632/oncotarget.8029)
Supplement: Supplementary file 1 [file oncotarget-07-20869-s001.pdf]

# Geminin overexpression-dependent recruitment and crosstalk with mesenchymal stem cells enhance aggressiveness in triple negative breast cancers

## Supplementary Materials

### Supplementary Figure S1A: Fold change in expression of indicated proteins normalized to tubulin and compared to values found in HME cells

| | HME | iGem9 | MDA-MB-231 | MDA-MB-468 | BT-549 |
|---|---|---|---|---|---|
| Geminin | 1 | 4.92 | 5.11 | 6.21 | 5.42 |
| HMGB1 | 1 | 1.07 | 1.02 | 1 | 1.02 |
| c-Abl | 1 | 4.62 | 5.42 | 5.34 | 2.83 |
| CBP | 1 | 0.94 | 0.91 | 1.05 | 1.12 |

### Supplementary Figure S1B: Fold change in expression of indicated proteins normalized to H2B and compared to values found on iGem9 $G_2$/M cells chromatin

| | G2/M | M/G1 | G1/S |
|---|---|---|---|
| Geminin | 1 | 1 | 0.12 |
| HMGB1 | 1 | 1.72 | 0.11 |
| c-Abl | 1 | 1.01 | 0.42 |
| CBP | 1 | 0.72 | 0.36 |
